# Community and Individual Education Influences on Sexual and Reproductive Health Knowledge in Uganda: A Human Capital and Social Learning Perspective

**DOI:** 10.1007/s11113-025-09958-y

**Published:** 2025-06-17

**Authors:** Stephanie Chamberlin, Leah Pauline, Patrick M. Krueger

**Affiliations:** 1 Department of Population Health Sciences, University of Wisconsin-Madison School of Medicine and Public Health, 610 Walnut Street, 707 WARF Building, Madison, WI 53726 USA; 2 Department of Health and Behavioral Sciences, University of Colorado Denver, Campus, Box 188 P.O. Box 173364, Denver, CO 80217-3364 USA

**Keywords:** Sexual and reproductive health, HIV, Contraception, International education, International development, Social learning, Sub-Saharan Africa, Human capital, Health knowledge

## Abstract

Higher levels of both individual education and community education may facilitate improved sexual and reproductive health knowledge, but our understanding of this relationship in sub-Saharan Africa remains limited. Drawing on human capital and social learning theories, and Demographic Health Survey data from Uganda, we examine the independent and interactive associations between individual and community education and two outcomes—HIV prevention knowledge and knowledge of different contraceptive methods—including differences by gender. Consistent with human capital and social learning theories, results from multilevel regression models show that both individual education and community education levels are independently and positively associated with more accurate sexual and reproductive health knowledge. Further, in support of the idea that human capital and social learning theories work in tandem, we find that the association between individual education and HIV knowledge is stronger in less educated communities, and grows weaker as community education increases, for both men and women. Similarly, for men, but not women, the association between individual education and contraceptive knowledge is stronger in less educated communities and weaker as community education increases. Among women, individual education was strongly and positively associated with contraceptive knowledge, an association that varied little across more or less educated communities. Our findings suggest that policy makers should consider community education levels when developing priorities for sexual and reproductive health knowledge interventions.

**Supplementary Information:**

The online version contains supplementary material available at 10.1007/s11113-025-09958-y.

## Introduction

Formal education is a determinant of improved sexual and reproductive health outcomes in sub-Saharan Africa, such as reduced rates of unintended pregnancy and HIV infection (Blackstone et al., 2017; Psaki et al., 2019). Many in the field point to the potential influence of formal education (i.e., schooling) on improved sexual and reproductive health *knowledge* as a theoretical explanation for downstream relationships between education and sexual and reproductive health outcomes. Yet, our empirical understanding of the influence of formal education on sexual and reproductive health knowledge remains superficial (Agüero & Bharadwaj, 2014; Basu, 2002; Psaki et al., 2019; Tsai & Venkataramani, 2015). Important questions remain about whether, how, and for whom formal education influences the acquisition of sexual and reproductive health knowledge—questions directly relevant to the Sustainable Development Goals (Sustainable Development Fund, 2015). Our study addresses these questions and informs efforts to empower individuals to achieve their sexual and reproductive health goals through improved sexual and reproductive health knowledge, contributing to reductions in pervasively high rates of unintended pregnancy (8–10%) and HIV prevalence (6%) in the southern and eastern African region (Bearak et al., 2022; UNAIDS, 2022). This study offers insight about how limited education resources can be targeted to achieve the greatest health benefits for the population. Currently, fewer than two-thirds of children in the region complete primary schooling (United Nations, 2023), and estimates suggest that achieving educational system capacity for universal enrollment would require doubling of the proportion of GDP currently allocated to education (Lewin, 2020). In Uganda, a setting where education access and resources are limited, we examine the sexual and reproductive health knowledge benefits from formal education among individuals and within communities.

Human capital perspectives—defined as people’s skills, knowledge, and experiences that can be harnessed to achieve goals—suggest that schooling can provide individuals with direct instruction on sexual and reproductive health, the development of skills (e.g., literacy and critical thinking) to seek out and discern accurate information, and greater access to health care and employment settings where knowledge may be disseminated (Baker et al., 2011; Mirowsky & Ross, 1998). At the same time, social learning and diffusion theories posit that sexual and reproductive health knowledge can be disseminated and normalized through organic social interactions and observations in formal and informal spaces, potentially among individuals with heterogenous educational backgrounds (Behrman et al., 2002; Montgomery & Casterline, 1996). These two perspectives, while suggesting different mechanisms, may work together—the knowledge and skills fostered by formal education may improve the accuracy of sexual and reproductive health knowledge that moves through communities via informal social learning. Indeed, individuals with less education may benefit from the sexual and reproductive health knowledge circulating among others in their social networks who have more education.

Our study examines these dynamic processes through three aims, using Demographic Health Survey Data from Uganda. First, we examine whether individual education *and* average levels of education in communities are separately associated with sexual and reproductive health knowledge. Second, we examine whether the average levels of education in communities modifies the association between individual education and sexual and reproductive health knowledge. Third, we examine whether these associations differ for men and women, thereby advancing prior research that focuses primarily on women. The gendered nature of schooling, health care, employment, and social norms in the region suggests that the association between education and sexual and reproductive health knowledge may vary by gender.

To achieve these aims, we examine two types of sexual and reproductive health knowledge—HIV and contraception—giving us greater insight into the social processes that may differentially shape educational associations with both types of knowledge. We specifically assess knowledge about HIV prevention and transmission, and awareness of various contraceptive method options. Each type of knowledge may be acquired in distinctive ways, suggesting that there may be unique relationships with individual and community education for these different types of knowledge. Knowledge about contraceptive options is closely tied to available reproductive health services and contraceptive technologies in a community, potentially limiting awareness and conversation about unavailable methods (Marcell et al., 2022; Skiles et al., 2015). Moreover, conversations around contraception may be more prevalent among women than men (Marcell et al., 2022; Watkins & Danzi, 1995). By comparison, HIV knowledge is likely shared informally through discussions about commonly held beliefs about how the virus is transmitted, even if no one attends a clinic, and discussions of HIV prevention are equally likely to occur among men and women (Behrman et al., 2003; Coursey et al., 2023). By using these two different comprehensive measures of sexual and reproductive health knowledge, we provide depth and breadth to our understanding of relationships between education and sexual and reproductive health knowledge.

We advance existing literature on population-level associations between formal education and sexual and reproductive health knowledge in sub-Saharan Africa, in three ways. First, we directly model the link between individual education and comprehensive contraceptive and HIV knowledge. Existing work largely examines the link between education and contraception use or unintended fertility, while theorizing (but not measuring) contraceptive knowledge as a mediating pathway (Apanga et al., 2020; Blackstone et al., 2017; Emina et al., 2014). The few papers that directly examine the links between education and HIV knowledge demonstrate a positive association (Agüero & Bharadwaj, 2014; De Walque, 2009; Glick & Sahn, 2007). Second, to our knowledge, we undertake the first examination of the relationship between community education and individuals’ sexual and reproductive health knowledge. Prior studies largely examine the association between community education levels and reproductive health outcomes, rather than knowledge itself (Benefo, 2006; Kravdal, 2002; Stephenson et al., 2007). The small but rich literature that examines the dissemination of sexual and reproductive health knowledge through social learning via social networks, does not consider the role of formal education in these processes (Behrman et al., 2003; Helleringer & Kohler, 2005; Kohler et al., 2000; Paek et al., 2006). Third, there is only one other study on the association between individual education and either contraceptive or HIV knowledge among men in the region that we know of (Glick & Sahn, 2007)—even though men play an important role in both HIV transmission and contraception (Marcell et al., 2022).

## Background

### Individual Education

Human capital theories suggest that more highly educated Ugandans should have more complete and accurate sexual and reproductive health knowledge when compared to those with less education, for three reasons. First, education supports the development of cognitive skills for processing and remembering written and verbal information about different methods of pregnancy and HIV prevention (Bloom, 2007; Smith-Greenaway, 2012). In Uganda, sexual and reproductive health information is commonly disseminated through community-based health promotion campaigns via local gatherings, radio programs, and fliers or posters (often in English). Higher levels of education allow individuals to more easily read and understand information on the internet, pamphlets, and/or posters that outline different contraceptive methods and HIV prevention strategies (Jukes et al., 2008; Yusuf et al., 2020). These skills also allow individuals to weigh the validity of information about sexual and reproductive health risks that are provided by radio or TV campaigns, medical professionals, colleagues, acquaintances, friends, and family members (Agüero & Bharadwaj, 2014; Mirowsky, 2017).

Second, education provides access to diverse social environments that may result in more opportunities to acquire more complete and accurate sexual and reproductive health information. More educated individuals in sub-Saharan Africa are more likely to seek and access quality health services, increasing their exposure to accurate health information (Chama-Chiliba & Koch, 2013; Larsson & Stanfors, 2014). Schools in the region also directly provide information about health and how to access health services, as well as connection to social networks of peers and teachers where sexual and reproductive health knowledge may be exchanged informally (Grant, 2015; Jukes et al., 2008). Finally, education opens doors to different kinds of employment in the formal economy (Cutler & Lleras-Muney, 2006), interacting with others with higher levels of education—opportunities often limited in rural areas of Uganda. Such employment can provide new exposure to additional health knowledge, often in new settings (Adepoju, 2000).

#### Hypothesis 1

*Individuals with more education will have more accurate sexual and reproductive health knowledge than those with less education*.

### Community Education

Living in a more educated community may enhance sexual and reproductive health knowledge for more and less educated individuals alike. This is consistent with other research in the region showing that higher average level of education in a community is linked with better maternal and child health outcomes, increased contraceptive use, and lower fertility, even after accounting for individuals’ education levels (Andrzejewski et al., 2009; Benefo, 2006; Kohler et al., 2000; Kravdal, 2002; Smith-Greenaway, 2017; Stephenson et al., 2007; Wasswa et al., 2021).

Extending the idea that more educated individuals will have greater sexual and reproductive health knowledge, communities with more highly educated residents are likely to have more accurate sexual and reproductive health knowledge on average. In Uganda, communities where education is more prevalent are also likely to have more schools and health institutions where more accurate sexual and reproductive health knowledge is disseminated formally. For HIV, billboards, radio, and TV ads regarding HIV prevention are likely more prevalent in more educated areas (Slutkin et al. 2006; Nalwadda et al., 2011). For contraception, the range of available contraceptive methods is quite variable across communities, and those communities with more education are also more likely to have a greater variety of contraceptive options available to them, increasing the likelihood that residents will have more exposure to a greater number of available methods. Thus, we expect that information about issues like contraception and HIV prevention circulating in highly educated communities will be more complete, more accurate, and more accessible for all community members.

Social learning theories further recognize that individuals simply living in a context with greater sexual and reproductive health information are likely to absorb that information through informal processes such as observation, hearsay, casual conversation, or common exposure to media and information campaigns. Indeed, such social learning processes have been well documented in the eastern African region (Bandura, 2001; Behrman et al., 2002; Sweya et al., 2016). As such, we expect as the average level of education increases in a community, so will the prevalence of more complete and accurate sexual and reproductive health knowledge.

#### Hypothesis 2

*Communities that have higher average levels of education will be associated with more accurate sexual and reproductive health knowledge than communities that have lower average levels of education*.

### Intersection of Individual and Community Education

Social learning theories recognize that individuals talk to and learn from the HIV and pregnancy prevention experiences of other members of the community (Bandura, 2001). While schools and health centers formally disseminate sexual and reproductive health knowledge in Uganda, more routine interactions are likely to account for a larger share of social learning in the region (Montgomery & Casterline, 1996). Contraception and HIV transmission are topics of formal and informal conversation in a variety of settings in eastern Africa, potentially shaping the information and norms surrounding sexual and reproductive health in Ugandan communities (Rutenberg & Watkins, 1997). Women who sit together in the waiting rooms of local health clinics can share information about their contraceptive choices. Indeed, health clinics are a common gathering place for women and mothers in Uganda. Men in the region who frequently socialize in areas like ‘beer huts,’ informal soccer matches, or at bus stops are known to share information about HIV risk and prevention in those settings (Coursey et al., 2023; Watkins, 2004). While somewhat taboo in larger public spaces, friends and family share examples of HIV transmission and prevention strategies from their social circles, or discuss their preferences for different contraceptive methods in direct conversation in the region (Bhushan et al., 2021; Watkins & Danzi, 1995). As such, sexual and reproductive health knowledge may be shared within communities where people live and work, across groups with different education levels.

Social learning theory further suggests that community levels of education may modify the association between individual education and sexual and reproductive health knowledge. Communities and social networks within communities are a powerful resource in Uganda—and in low resource settings more broadly—for acquiring various types of information. However, the knowledge circulating in eastern African communities is known to be of variable completeness and accuracy, and individuals may be unevenly susceptible to adopting the knowledge circulating in a community (Bhushan et al., 2021; Sedlander et al., 2021). Individuals with more education may have access to more accurate sexual and reproductive health knowledge through work and schooling networks, irrespective of the ideas circulating in their respective home communities (Cutler & Lleras-Muney, 2010; Lawrence, 2017). Further, more educated individuals can draw on their cognitive resources to identify complete and accurate information, and question or reject less complete or inaccurate information, regardless of the context of their home community (Mirowsky & Ross, 2005). In contrast, less educated individuals may be more likely to adopt the prevailing sexual and reproductive health knowledge circulating in a community. Adults with less education have had fewer opportunities to cultivate literacy and critical thinking skills that can help guard against inaccurate knowledge, and they have less exposure to accurate sexual and reproductive health knowledge disseminated in school and health care settings. As such, less educated adults in Uganda may benefit from living in more educated communities where more accurate knowledge is likely in wider circulation, and where the institutions (e.g., health care settings, workplaces, mass media, social services) can support the dissemination of those ideas.

#### Hypothesis 3

*The association between individual education and sexual and reproductive health knowledge will be stronger in less educated communities and weaker in more educated communities*.

### Gender, Education, and Sexual and Reproductive Health Knowledge

Gendered divisions of labor, household responsibilities, and health care use in eastern Africa suggest that social learning processes are likely to play out in distinct ways for men and women in the region. At the same time, we know that communities are not constructed along gender lines and that men and women interact and influence each other every day. Nonetheless, research on sexual and reproductive health in sub-Saharan Africa focuses predominately on women. We extend prior work by including both men and women in our individual and community measures. Since the 1994 Conference on Population and Development in Cairo, interest in men’s sexual and reproductive health and the role that men play in women’s sexual and reproductive health has greatly expanded (Mundigo, 2000). Men are integral family and community members who converse with their partners and their peers to gain and share sexual and reproductive health knowledge and influence sexual and reproductive health decisions (Dodoo and Frost, 2008; Thummalachetty et al., 2017).

Men’s education may play an important role in shaping their sexual and reproductive health attitudes and practices, which suggests that their education may also influence the sexual and reproductive health knowledge of their communities. While men’s role in sexual and reproductive health has gained attention (Chan & Tsai, 2018; Glick & Sahn, 2007), most empirical work looking at the relationship between community education and individual sexual and reproductive health in sub-Saharan Africa still excludes men from aggregate community measures entirely (Kravdal, 2002; Smith-Greenaway, 2017; Stephenson et al., 2007). Thus, we are missing information on half of the community and limiting our understanding of the interactions between men and women in communities and their impact on sexual and reproductive health. Our approach expands knowledge about men’s education and sexual and reproductive health knowledge more broadly, and increases our understanding about the role of education within the whole community.

Several key factors lead us to believe that Ugandan men’s individual education will strongly and positively influence their sexual and reproductive health knowledge, regardless of the education level in their community context. First, compared to women, men in Uganda are more likely to travel outside of their geographic home community for work, expanding their exposure to an array of sexual and reproductive health information from resources external to the home community. Second, men in east Africa may be less likely than women to discuss sexual and reproductive health topics—themes that are considered the social domain of women—in their communities (Behrman et al., 2002). Third, the fact that men in the region less frequently access health care and are often peripheral to family planning services means that they are also less likely than women to benefit from the information disseminated through these community resources (Hardee et al., 2017; Yeatman et al., 2018). Ugandan men’s relatively limited formal and informal interactions around sexual and reproductive health topics suggests that they may rely more on their individual education to access—and the cognitive skills to discern the quality of—sexual and reproductive health information. As a result, men in Uganda with greater individual education may be likely to obtain more accurate sexual and reproductive health knowledge in communities with lower and higher education alike.

#### Hypothesis 4a

*There will be a strong, positive association between individual education and sexual and reproductive health knowledge for men across community contexts*.

In contrast to men, women in east Africa routinely engage with the local health care system and frequently attend health education events in the community (Hardee et al., 2017; Yeatman et al., 2018). Women are also more likely than men to discuss contraceptive choices and sexual health topics with each other, given their reproductive and family caregiver roles within their communities (Mosha et al., 2013). Compared to men, women in rural Uganda typically have fewer opportunities for interactions outside of their local communities due to more limited work-related travel, suggesting that their home community’s education levels may strongly influence the sexual and reproductive health information available to them (Foley et al., 2022; Glick & Sahn, 2007). Ugandan women living in more educated areas will likely benefit from the more accurate sexual and reproductive health information circulating in their communities, leading to similarly high levels of sexual and reproductive health knowledge for women across the educational spectrum. But, in lower educated Ugandan communities, where sexual and reproductive health information is either more difficult to access or less accurate, less educated women may be prone to lower levels of accurate sexual and reproductive health knowledge. In contrast, the knowledge, skills, and resources available to more educated Ugandan women in less educated areas may buffer them from the more limited or inaccurate sexual and reproductive health knowledge in circulation. Thus, for women in Uganda, the positive relationship between individual education and sexual and reproductive health knowledge will be stronger in communities with lower average education levels.

#### Hypothesis 4b

*For women, the association between individual education and sexual and reproductive health knowledge will be weak in more educated communities and strongly positive in less educated communities*.

### Study Setting

Uganda’s social and policy context provides a useful case for our study for four reasons. First, Uganda was among the first countries in the region to implement the nationwide policy of universal primary education in 1997 and has continued to promote education access since then (Miles & Singal, 2010). Yet, increased educational access has yielded mixed results for economic and health outcomes, placing Uganda at the center of an international debate about the value and quality of mass education (Chudgar et al., 2019; Datzberger, 2018). Although education access has expanded dramatically in the past three decades, in 2016, 16% of the population age 6 and over had never entered schooling with a median of 3.6 years of schooling (DHS Program STATcompiler, 2020). Second, like many countries in the region, Uganda has had mixed success with family planning and HIV prevention interventions. Access to contraceptive methods remains limited in many areas of the country, with pervasively high rates of unmet contraceptive need (Guttmacher Institute, 2017). Despite many successes in curbing the HIV epidemic in the 1980s and 90s using intensive local-level health education interventions (Green et al., 2006), the country has seen a resurgence in HIV incidence, stigma, and mortality in the past decade (Chan et al., 2015; Tsai & Venkataramani, 2015). This makes disseminating accurate HIV prevention and contraceptive knowledge a critical public health priority.

Third, like other countries in Sub-Saharan Africa, Uganda has a long history of efforts to decentralize government administration to the local level with an increased emphasis on the ‘localization’ of education and health interventions (Namukasa & Buye, 2007; Tashobya et al., 2018). Thus, community-level analyses like the ones we conduct in this study, can contribute to efforts to plan for, invest in, and evaluate the effects of educational and health investments that jointly consider community context, individual need, and national resource distribution. Fourth, entrenched gender dynamics in Uganda mean that women’s education and health care access, and social and economic lives are often distinct from men’s (Mukasa et al., 2024; Paek et al., 2006). Our assessment of differences by gender is particularly pertinent in the Ugandan context. Finally, the international policy discourses, health and education challenges, and gender dynamics that shape Ugandan social policy are also prominent in most eastern African countries—potentially making our findings applicable for the broader region.

## Data and Methods

### Data

We use cross-sectional data from the 2016 Uganda Demographic Health Survey (DHS) to test our hypotheses. The DHS is a nationally representative survey that was carried out approximately every five years across sub-Saharan Africa. The 2016 Uganda DHS contains a random sample of 15,280 households with completed interviews stratified within 696 selected geographic enumeration areas (referred to as clusters), the primary sampling unit for the survey, in 34 regions. Consistent with pior studies using DHS data to examine community-level effects, we define a community based on the geographic boundary for each cluster, typically representing a village in rural areas or city block in urban areas (Kravdal, 2002; Smith-Greenaway, 2017). On average, a cluster contained 123 households in urban areas and 85 households in rural areas (Uganda Bureau of Statistics, 2018). Survey participants included all eligible women from 15 to 49 years old within a sampled household (*N* = 18,506), and men 15–54 years old from every third household (*N* = 5336), with response rates > 95% (Uganda Bureau of Statistics, 2018). After excluding respondents with missing data or communities with data on one or fewer men, our final sample size includes 5327 men and 18,316 women from 15,121 households across 686 clusters (i.e., communities).

DHS surveys are ideal for multilevel analyses as they utilize standardized questions within a similar time frame for each community. We draw on individual surveys that provide sociodemographic characteristics and the key study variables for education and sexual and reproductive health knowledge responses, and household surveys for indicators of household wealth and the characteristics of the community wherein each household resides. The DHS survey was administered by trained interviewers in one of the seven main languages in Uganda, as appropriate for the respondent. Further detailed information on the Uganda 2016 DHS sample design and data collection can be found at: www.dhsprogram.com (ICF, 2018; Rutstein & Rojas, 2006).

### Variables

We use comprehensive measures of HIV prevention and contraceptive options, assessed separately, as the outcomes in our analyses. Our approach contrasts with the commonly assessed ‘basic’ sexual and reproductive health knowledge—a simple dichotomous measure of awareness of the existence of HIV or family planning—an awareness that is nearly universal in the region (Agyei & Migadde, 1995; Blackstone et al., 2017; Chudgar et al., 2019). The comprehensive measures we use better assess an understanding of different modes of HIV transmission and awareness of various contraceptive options—knowledge that is less prevalent in the region and more likely to influence HIV prevention behaviors and contraceptive use (Mbugua & Karonjo, 2018; Nsubuga et al., 2015; Oonyu, 2020).

The DHS contains seven survey items measuring knowledge of HIV prevention and transmission risk (e.g., “Is it possible for a healthy-looking person to have HIV?”), with yes/no/don’t know response options (survey questions are listed in online appendix Fig. S1), which are similarly used in related research (Teshale et al., 2022). We recode each item with 1 for the correct answer and 0 for any other response. We then sum the items to calculate the total scale score (ranging from 0 to 7) for comprehensive HIV prevention knowledge—a commonly used knowledge index in DHS reports (Chan & Tsai, 2018). The DHS also asks whether respondents have ever heard of eleven different types of modern contraception available in Uganda: pill, IUD, injection, male and female condom, male and female sterilization, implant, emergency contraception, standard days method, and lactation (survey questions are listed in online appendix Fig. S2). We code knowledge of a given method as 1 and any lack of knowledge as 0, and then sum the items to calculate the total score of contraceptive awareness (0–11).

Our key predictors include individual education, community education, and gender. Individual education is a continuous measure of the total years of education completed, based on self-reports of the highest grade (i.e., year) completed within the highest level of schooling completed (i.e., none, primary, secondary, or tertiary). For example, in Uganda, if a person reports their highest level of education as “secondary schooling” and four years of education completed at that level, we calculate that they attained 11 total years of formal schooling (i.e., 7 years of primary + 4 years of secondary). We calculate community education level as the mean of all men’s and women’s individual years of education within a community. For ease of interpretation, we mean-center our community education variable in all models. To assess the extent to which a community’s education modifies the association between individual education and sexual and reproductive health knowledge, some of our models include an interaction term between the individual and community education variables. We include a binary measure of gender (men coded 1 vs. women coded 0) to control for the ways in which gendered societal interactions may directly influence exposure to sexual and reproductive health information. Further, some models include a three-way interaction term (i.e., individual education × community education × gender) to test whether gender modifies the interaction of community and individual education.

We adjust for individual-level covariates including age, marital status, wealth, and employment. We measure age continuously, centered at the mean, to capture the potential difference in sexual and reproductive health knowledge between older and younger persons (Teshale et al., 2022). Marital status influences sexual and reproductive activities and exposures to different health issues. Thus, we control for a binary measure of whether an individual has ever been married (coded 1) or not (coded 0)—where responses of “currently married” or “previously but not currently married” are combined as “ever married.” We include household wealth as a potential confounder, which has been linked to both greater educational and health resource access (Filmer & Pritchett, 1999; Lartey et al., 2016). Our measure of wealth is drawn from the five-point index of wealth, from poorest (= 1) to richest (= 5), constructed by the DHS using a principal component analysis of household and individual assets and standardizing the distribution of wealth scores (Rutstein et al., 2004). Finally, employment that requires interactions outside of the home is a potential source of health information exposure. Thus, we control for a binary measure of any form of paid (cash) employment (coded 1) versus ‘no employment’ or ‘in-kind’ payment in the past 12 months (coded 0).

We also considered the need to adjust for exposure to other communities or social groups by adjusting for travel and internet use. Both of these exposure variables could be hypothesized as confounding factors, as their association with education at the individual and community levels is complex in Uganda, and both provide access to a wide variety of accurate and inaccurate health information. It is also possible that these factors may act as mediators between education and sexual and reproductive health knowledge. In the absence of longitudinal data about the temporal ordering of these variables, and given the risk of over-control bias (Elwert & Winship, 2014), we exclude them from our main analyses, but include them in sensitivity analyses in the online appendix. In Tables S2 and S3, we provide models controlling for a binary measure of any travel involving at least one night away from home in the past 12 months (coded 1) versus none (coded 0), and a binary measure of weekly or greater internet use (coded 1) versus less than weekly internet use (coded 0).

We adjust for community-level covariates including difficulty accessing health services, prevalence of employment, age-structure, community wealth, and rurality. Resources such as non-governmental organizations, health services, social services, community centers, and schools tend to cluster together in communities, providing more access to both formal schooling and more accurate sexual and reproductive health knowledge and suggesting a potentially confounding relationship (Benefo, 2006; Smith-Greenaway, 2012). We adjust for this by including barriers to accessing health services within a community, which we calculate as the proportion of women^1^ in a community who reported that distance to a health facility would be a ‘big problem’ if they needed medical treatment or advice. This allows us to control for the possibility that simply living in a more highly resourced community will improve access to sexual and reproductive health knowledge. For each DHS cluster, we also control for the percent employed, the average age of the population, the percent of households that rank below ‘average’ on the aforementioned household wealth index, and whether the household was in a rural community.^2^ We use the DHS-constructed binary measure of rural (coded 1) versus urban (coded 0), where urban is defined in concordance with the Uganda 2014 Census (2016).

### Analysis

We examine the associations between individual and community education levels and sexual and reproductive health knowledge using multilevel linear regression models that allow us to parse within- and between- community variation in sexual and reproductive health knowledge, while estimating appropriate standard errors given the non-independence of individuals living within the same community. We estimate five models for each sexual and reproductive health knowledge outcome. Model 1 includes one fixed effect (the intercept), a random intercept (i.e., the variance of the community intercepts around the population average intercept), and a residual variance (i.e., the variance of each respondents’ intercepts around their community-specific intercepts). The random intercept and the residual variance allow us to calculate the intra-class correlation, which summarizes the share of the variability in the outcome that occurs within communities.

Models 2–4 include additional fixed effects. Model 2 includes covariates for individual education, community education, and gender—the key predictors in our analyses. Model 3 further controls for additional individual and community characteristics. Model 4 includes the cross-level interaction between individual and community education to test whether the relationship between sexual and reproductive health knowledge and individual education is moderated by the level of education in the community. Model 5 includes a three-way interaction between gender, individual education, and community education to test whether the results from Model 4 differ across gender. Models 2–4 also include random slopes for education (i.e., the variance between each community’s slope for education around the population average slope for education), gender (i.e., the variance of each community’s slope for gender around the population average slope for gender), and covariances between the random slope for education and the random intercept, the random slope for gender and the random intercept, and the random slope for education and the random slope for gender.

We combine men’s and women’s data to acquire all individual-level variables, and merged household data to obtain household weights for constructing multilevel weights. We first denormalize all survey weights to account for men’s under-sampling in the survey design, allowing us to pool men’s and women’s data to obtain a community level measure of education (ICF, 2018). Next we approximate two separate survey weights at the individual and then at the cluster (i.e., community) level as recommended by the DHS for appropriately weighting our multilevel data (Mahmoud Elkasabi, 2020). We incorporate these survey weights in all of our descriptive, bivariate, and multivariate analyses. We conduct all analyses in Stata version 18 (StataCorp, 2023).

## Results

Table 1 shows weighted means and percentages of our key variables, and tests for differences by gender. People knew an average of 8 out of the 11 types of modern contraceptive options, which was slightly higher for women (8.3) compared to men (8.0). Men and women reported high average levels of HIV knowledge at 6.1 on a 7-point scale. Individuals completed an average of 7.1 years of schooling, which was lower for women (6.3 years) than for men (7.4 years). Across communities, the average years of educational attainment within a community was 6.8 (before mean centering), just slightly lower than the average for individual education.

**Table 1:** Means and percentage distribution of DHS Uganda 2016 sample characteristics, weighted, by gender

	Total	Women	Men	*p*-value^a^
***Outcome variables***				
Contraceptive knowledge score (range = 0–11)	8.1	8.3	8.0	< 0.001
HIV knowledge score (range = 0–7)	6.1	6.1	6.1	0.128
***Key predictor variables***				
Individual education (years)	7.1	6.3	7.4	< 0.001
Community education (years)^b^	6.8	6.8	6.7	0.041
Gender (Male = 1, %)	–	–	–	–
***Individual-level control variables***				
Age (years)^b^	28.6	27.9	29.2	< 0.001
Marital status (Ever married = 1, %)	67.5	74.1	60.9	< 0.001
Wealth (quintile, %)				
Poorest	17.1	16.8	17.4	0.010
Poor	18.1	17.8	18.4	
Average	19.2	19.7	18.7	
Rich	21.0	22.0	20.0	
Richest	24.7	23.8	25.6	
Employment (Paid work in past year = 1, %)	62.7	57.7	67.7	< 0.001
***Community-level control variables***				
% Distance to clinic as a barrier in community	37.8	37.4	38.3	0.011
% Employed in community	60.0	59.8	60.0	0.487
Average of mean age in community	28.2	28.2	28.2	0.283
% Households in a community with less than average wealth	35.7	35.4	36.0	0.227
Rural household (Rural = 1, %)	74.3	73.5	75.1	0.033
***Unweighted N***	23,643	18,316	5,327	–

^a^ Differences between men and women were tested using bivariate logistic regression for continuous variables and Pearson’s chi-square for categorical variables

^b^ Mean centered in multivariate models

### HIV Knowledge

Table 2 shows multilevel linear regression results for HIV knowledge. Model 1 includes the intercept, the random intercept variance, and the residual variance, and allows us to examine variation in HIV knowledge within and between communities, with a population average intercept of 6.053. The random intercept shows that the variance in the distribution of community-level intercepts for HIV knowledge around the population average intercept for HIV knowledge is 0.147. The residual variance shows that the distribution of community residents’ HIV knowledge around their community average intercepts for HIV knowledge is 1.090. We use these two variances to calculate the intra-class correlation (ICC = 0.147/(1.090 + 0.147) = 0.119), which shows that 11.9% of the variance in HIV knowledge occurs between community clusters.

**Table 2:** Multilevel linear regression models of knowledge of HIV prevention and transmission, fixed and random effects (coefficients with standard errors in parentheses)

	Model 1^a^	Model 2^b^	Model 3^c^	Model 4^d^	Model 5^e^
**Fixed effects**					
Individual education		0.065^***^ (0.003)	0.067^***^ (0.003)	0.065^***^ (0.003)	0.061^***^ (0.003)
Community education^f^		0.041^***^ (0.006)	0.028^**^ (0.010)	0.068^***^ (0.013)	0.057^***^ (− 0.014)
Gender (Male = 1)		− 0.091^***^ (0.023)	− 0.078^**^ (0.024)	− 0.079^***^ (0.024)	− 0.107^*^ (0.047)
***Interaction terms***					
Individual education × Community education^f^			–	− 0.006^***^ (0.001)	− 0.005^***^ (0.001)
Individual education × Gender			–	–	0.008 (0.005)
Community education^f^ × Gender			–	–	0.055^*^ (0.026)
Individual education × Community education^f^ × Gender			–	–	− 0.003 (0.002)
***Individual-level control variables***					
Age^f^			0.006^***^ (0.001)	0.006^***^ (0.001)	0.006^***^ (0.001)
Marital status (Ever married = 1)			0.170^***^ (0.026)	0.170^***^ (0.026)	0.170^***^ (0.026)
Wealth (quintile)					
Poorest (ref)			–	–	–
Poor			0.078^*^ (0.034)	0.077^*^ (0.034)	0.076^*^ (0.034)
Average			0.127^**^ (0.041)	0.125^**^ (0.041)	0.125^**^ (0.041)
Rich			0.104^*^ (0.045)	0.103^*^ (0.045)	0.103^*^ (0.045)
Richest			0.191^***^ (0.052)	0.191^***^ (0.052)	0.191^***^ (0.052)
Employment (Paid employment in past year = 1)			0.016 (0.022)	0.016 (0.022)	0.089 (0.088)
***Community-level control variables***					
% Distance to clinic as a barrier in community			0.158^**^ (0.054)	0.154^**^ (0.054)	0.154^**^ (0.054)
% Employed in community			0.083 (0.088)	0.087 (0.088)	0.088 (0.088)
Mean age in community			− 0.0003 (0.007)	− 0.0006 (0.007)	− 0.0006 (0.007)
% Households with less than average wealth in community			− 0.049 (0.070)	− 0.045 (0.070)	− 0.053 (0.069)
Rural household (Rural = 1)			0.007 (0.037)	0.002 (0.037)	0.005 (0.0367)
*Intercept*	6.053^***^ (0.014)	5.700^***^ (0.022)	5.360^***^ (0.197)	5.388^***^ (0.196)	5.403^***^ (0.197)
**Random effects**					
Random intercept	0.147^***^ (0.015)	0.249^***^ (0.016)	0.242^***^ (0.015)	0.237^***^ (0.015)	0.240^***^ (0.015)
Random slope for individual education		0.004^***^ (0.0003)	0.004^***^ (0.0003)	0.003^***^ (0.0003)	0.0034^***^ (0.0003)
Covariance between the random slope for education and the random intercept		− 0.024^***^ (0.002)	− 0.023^***^ (0.002)	− 0.022^***^ (0.002)	− 0.023^***^ (0.002)
Random slope for gender		0.395^***^ (0.033)	0.393^***^ (0.033)	0.393^***^ (0.033)	0.380^***^ (0.029)
Covariance between the random slope for gender and the random intercept		− 0.038^*^ (0.015)	− 0.036^*^ (0.016)	− 0.043^**^ (0.015)	− 0.041^**^ (0.015)
Covariance between the random slope for education and the random slope for gender		− 0.009^***^ (0.002)	− 0.009^***^ (0.002)	− 0.009^***^ (0.002)	− 0.008^***^ (0.002)
Residual variance	1.090^***^ (0.027)	0.926^***^ (0.025)	0.911^***^ (0.024)	0.911^***^ (0.024)	0.910^***^ (0.024)

^†^*p* < 0.10, ^*^p < 0.05, ^**^p < 0.01, ^***^p < 0.001

^a^ Model 1 contains no predictors, allowing for an examination of the average intercept for HIV knowledge and the variation in HIV knowledge across and within different communities

^b^ Model 2 tests Hypotheses 1 and 2: The associations between HIV knowledge and education at the individual and community levels, when controlling only for gender

^c^ Model 3 tests Hypotheses 1 and 2: The associations between HIV knowledge and education at the individual and community levels, when controlling for other individual and community factors

^d^ Model 4 tests Hypotheses 3: The moderating (i.e., spillover) effect of community education on the association between individual education and HIV knowledge, when controlling for other individual and community factors

^e^ Model 5 tests Hypotheses 4a and 4b: The gender difference in the moderating effect of community education on the association between individual education and HIV knowledge, when controlling for other individual and community factors

^f^ Variable is mean centered

Model 2 includes our three key predictors—individual education, community education, and gender—and provides a first test of the association between individual education (Hypothesis 1), community education (Hypothesis 2), and HIV knowledge. Each additional year of individual education is associated with a 0.065 point increase in the HIV knowledge score, when adjusting for community education and gender. Each additional year of community education is associated with a 0.041 point increase in the HIV knowledge score, when adjusting for individual education and gender. Finally, compared to women, men have a 0.091 point lower HIV knowledge score, when adjusting for individual and community education. Model 3 shows that the association between individual education and HIV knowledge is largely unchanged (0.067) after adjusting for individual and community covariates. Although adjusting for covariates partially explains the relationship between community education (0.028) and gender (-0.078) with HIV knowledge, the direction of these associations remains the same.

Model 4 further includes the interaction between individual education and community education, to test whether community education moderates the association between individual education and HIV knowledge (Hypothesis 3). We find that the association between individual education and HIV knowledge becomes weaker as community education increases. The coefficient for individual education shows that each additional year of education is associated with a 0.065 point increase in HIV knowledge, at the mean of community education (i.e., when the mean-centered community education variable is equal to 0) and when adjusting for all other variables. The coefficient for community education shows that a one-year increase in the average level of education in a community is associated with a 0.068 point increase in HIV knowledge, when individual education is equal to 0, and when adjusting for all other variables. The interaction term shows that the association between individual education and HIV knowledge becomes 0.0058 points less positive for each additional one-year increase in community education, when adjusting for the other variables in the model.

**Fig. 1:**
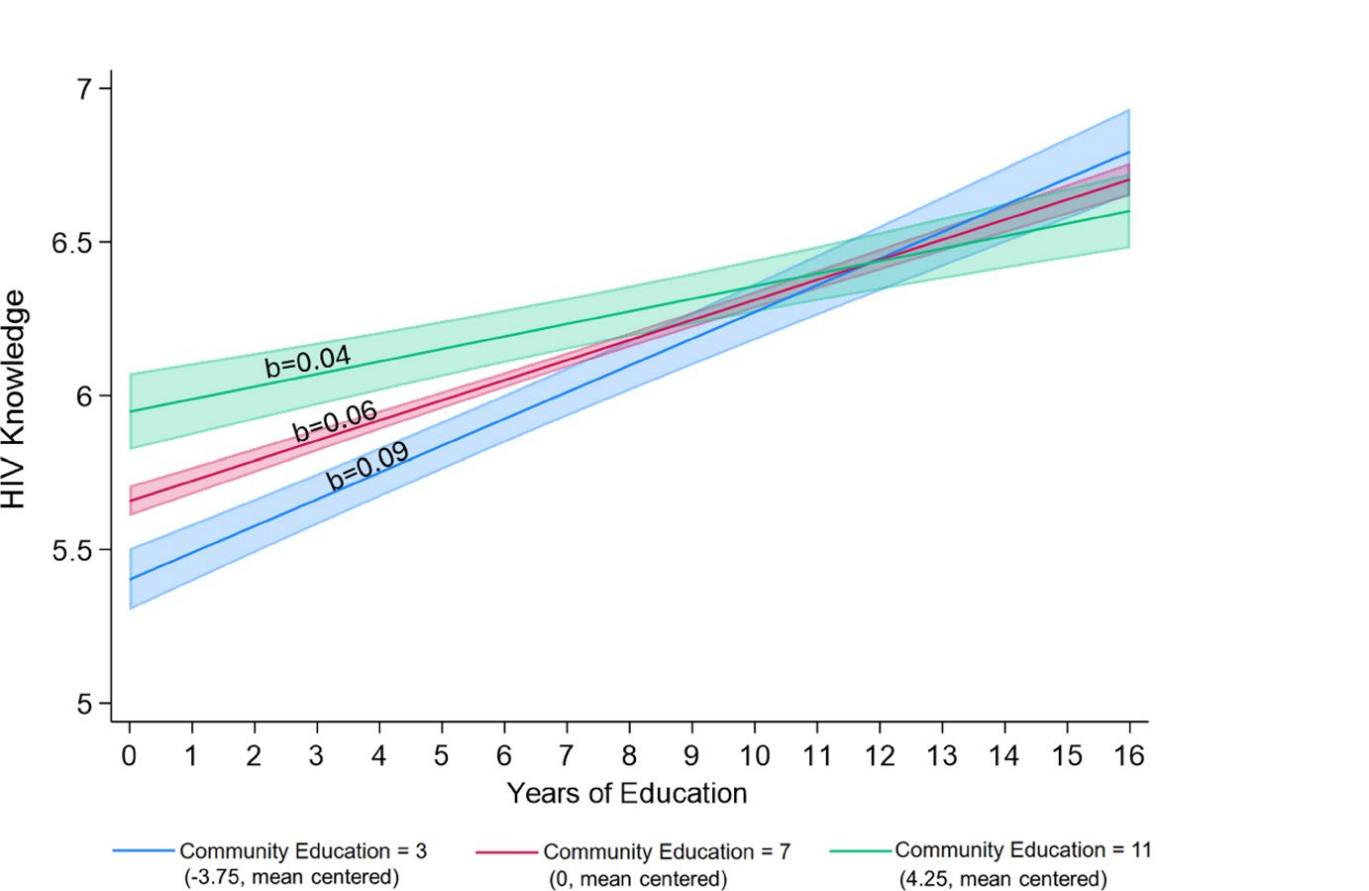
Predicted association between individual education and knowledge of HIV prevention and transmission in communities with 3, 7, and 11 years of education on average^a,b^. *Notes:* ^a^ Predicted margins with 95% confidence intervals are calculated based on results from Table 2, Model 4 at the mean of all control variables: gender, age, marital status, household wealth, paid employment, % distance to a health clinic as a barrier to care, % of community with paid employment, mean community age, % of community with below average wealth, and rural household. ^b^ Three years of education represents the end of lower primary schooling and approximately two standard deviations below the mean for community education. Seven years of education represents the end of upper primary schooling and is approximately the mean for community education. Eleven years of education represents the end of secondary schooling and approximately two standard deviations above the mean for community education

Figure 1 plots these interactions, with the individual education along the x-axis, HIV knowledge measured along the y-axis, and separate lines for different levels of community education. We consider 3, 7, and 11 years of community education (i.e., − 3.75, 0, and 4.25 when mean centered), which represent the end of lower primary schooling, upper-primary schooling, and secondary schooling, respectively, and roughly correspond with the community education mean (6.8) and two standard deviations above (11.0) and below (2.5) the mean—slopes are provided to demonstrate the gradient between individual education and HIV knowledge in each community segment. We include 95% confidence intervals (CIs). Figure 1 shows that the positive associations between individual education and HIV knowledge are stronger in communities with less education and weaken as community education increases. The magnitude of these effects is substantial. In communities with an average of 3 years of schooling (i.e., − 3.75 when mean centered), each year of education is associated with 0.09 (= 0.065 − 0.006 × − 3.75) additional points in HIV knowledge, or one full point increase in HIV knowledge for individuals with one versus 11 years of education. However, in communities with an average of 11 (i.e., 4.25 when mean centered) years of schooling, each additional year of education is associated with 0.04 (= 0.065 − 0.006 × 4.25) additional points in HIV knowledge; equating to less than half a point increase from those with one year of education compared to those who attain 11 years of education.

**Fig. 2:**
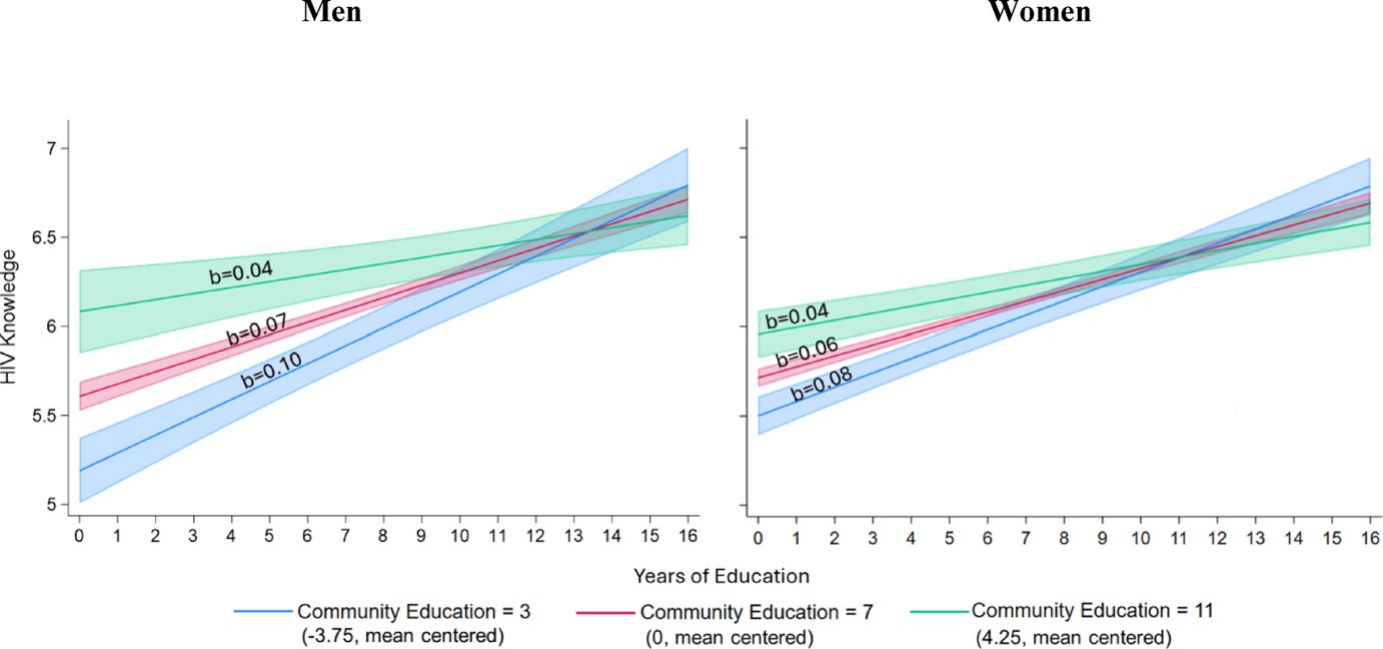
Predicted association between individual education and knowledge of HIV prevention and transmission for men and women in communities with 3, 7, and 11 years of education on average^a,b^. *Notes:* ^a^ Predicted margins with 95% confidence intervals are calculated separately for men and women based on results from Table 2, Model 5 at the mean of all control variables: age, marital status, household wealth, paid employment, % distance to a health clinic as a barrier to care, % of community with paid employment, mean community age, % of community with below average wealth, and rural household. ^b^ Three years of education represents the end of lower primary schooling and approximately two standard deviations below the mean for community education. Seven years of education represents the end of upper primary schooling and is approximately the mean for community education. Eleven years of education represents the end of secondary schooling and approximately two standard deviations above the mean for community education

Model 5 further includes two- and three-way interactions between individual education, community education, and gender to test whether the moderating effect of community education on the association between individual education and HIV knowledge from Model 4 differs for men and women (Hypotheses 4a and 4b). For each additional year of individual education there is a 0.061 increase in HIV knowledge, for women, at the mean years of community education, and when adjusting for all other variables in the model. The association between individual education and HIV knowledge among women becomes 0.005 points less positive for each additional 1-year increase in community education. For men, each additional year of individual education is associated with a 0.069 (= 0.061 + 0.008) increase in HIV knowledge, at the mean years of community education, and adjusting for all other variables. Further, among men, the association between individual education and HIV knowledge becomes − 0.008 (= − 0.005 + − 0.003) points less positive for each additional 1-year increase in community education. However, the three-way interactive effective with gender is not statistically significant at the 95% confidence level (*p* = 0.191), suggesting that the moderating effect of community education on the association between individual education and HIV knowledge does *not* vary by gender. To further interpret these associations from Model 5, we use Fig. 2, with separate panels for men and women. Each graph displays the associations between individual education and HIV knowledge for communities with 3, 7, and 11 years of average education (i.e., − 3.75, 0, and 4.25, when mean centered) for men and women respectively. The gradient of each slope is also provided in each panel, showing similar and steeper associations between individual education and HIV knowledge in less educated communities, and shallower associations between individual education and HIV knowledge in more educated communities for both men and women.

### Contraceptive Knowledge

Table 3 shows multilevel linear regression coefficients for contraceptive knowledge. Model 1 includes a population intercept of 8.067 and excludes all fixed effect predictors. There is a random intercept of 1.101 and a residual variance of 5.659, resulting in an ICC of 0.242 (= 1.101/(5.659 − 1.101))—indicating that approximately 24% of the variance in contraceptive knowledge occurs due to differences between communities.

**Table 3:** Multilevel linear regression models of knowledge of contraceptive methods, fixed and random effects (coefficients with standard errors in parentheses)

	Model 1^a^	Model 2^b^	Model 3^c^	Model 4^d^	Model 5^e^
***FixedEffects***					
Individual education		0.130^***^ (0.006)	0.161^***^ (0.006)	0.159^***^ (0.006)	0.142^***^ (0.006)
Community education^f^		0.065^***^ (0.018)	0.063^*^ (0.027)	0.100^**^ (0.032)	0.073^*^ (0.032)
Gender (Male = 1)		− 0.507^***^ (0.056)	− 0.406^***^ (0.055)	− 0.408^***^ (0.055)	− 0.592^***^ (0.108)
***Interactionterms***					
Individual education × Community education^f^			–	− 0.006^*^ (0.003)	0.0005 (0.003)
Individual education × Gender			–	–	0.035^**^ (0.013)
Community education^f^ × Gender			–	–	0.065 (0.054)
Individual education × Community education^f^ × Gender			–	–	− 0.013^*^ (0.006)
***Individual-level control variables***					
Age^f^			0.037^***^ (0.003)	0.037^***^ (0.003)	0.036^***^ (0.003)
Marital status (Ever married = 1)			1.542^***^ (0.065)	1.541^***^ (0.065)	1.541^***^ (0.065)
Wealth (quintile)					
Poorest (ref)			–	–	–
Poor			0.124^†^ (0.068)	0.123^†^ (0.068)	0.120^†^ (0.068)
Average			0.160^*^ (0.074)	0.159^*^ (0.074)	0.157^*^ (0.074)
Rich			0.261^**^ (0.087)	0.260^**^ (0.087)	0.260^**^ (0.087)
Richest			0.311^**^ (0.113)	0.311^**^ (0.113)	0.310^**^ (0.113)
Employment (Paid employment in past year = 1)			0.413^***^ (0.044)	0.413^***^ (0.044)	0.413^***^ (0.044)
***Community-level control variables***					
% Distance to clinic as a barrier in community			0.553^***^ (0.145)	0.549^***^ (0.145)	0.546^***^ (0.145)
% Employed in community			− 0.362 (0.241)	− 0.355 (0.241)	− 0.377 (0.241)
Mean age in community			0.0005 (0.018)	− 0.0001 (0.018)	− 0.0005 (0.018)
% Households with less than average wealth in community			− 0.292 (0.198)	− 0.288 (0.198)	− 0.340^†^ (0.197)
Rural household (Rural = 1)			0.135 (0.0895)	0.133 (0.0898)	0.162^†^ (0.091)
*Intercept*	8.067^***^ (0.037)	7.467^***^ (0.050)	5.728^***^ (0.545)	5.756^***^ (0.543)	5.861^***^ (0.543)
***Random effects ***					
Random intercept	1.101^***^ (0.079)	1.517^***^ (0.101)	1.183^***^ (0.085)	1.177^***^ (0.085)	1.201^***^ (0.086)
Random slope for individual education		0.020^***^ (0.002)	0.016^***^ (0.001)	0.016^***^ (0.001)	0.016^***^ (0.001)
Covariance between the random slope for education and the random intercept		− 0.143^***^ (0.011)	− 0.107^***^ (0.009)	− 0.107^***^ (0.009)	− 0.108^***^ (0.009)
Random slope for gender		2.225^***^ (0.152)	2.234^***^ (0.174)	2.236^***^ (0.174)	2.173^***^ (0.172)
Covariance between the random slope for gender and the random intercept		0.197^*^ (0.084)	0.127^†^ (0.074)	0.130^†^ (0.074)	0.120 (0.075)
Covariance between the random slope for education and the random slope for gender		− 0.050^***^ (0.011)	− 0.040^***^ (0.096)	− 0.040^***^ (0.097)	− 0.038^***^ (0.010)
Residual variance	5.659^***^ (0.115)	4.617^***^ (0.088)	3.655^***^ (0.070)	3.655^***^ (0.070)	3.651^***^ (0.070)

^†^*p* < 0.10, ^*^p < 0.05, ^**^p < 0.01, ^***^p < 0.001

^a^ Model 1 contains no predictors, allowing for an examination of the average intercept for contraceptive knowledge and the variation in contraceptive knowledge across and within different communities

^b^ Model 2 tests Hypotheses 1 and 2: The associations between contraceptive knowledge and education at the individual and community levels, when controlling only for gender

^c^ Model 3 tests Hypotheses 1 and 2: The associations between contraceptive knowledge and education at the individual and community levels, when controlling for other individual and community factors

^d^ Model 4 tests Hypotheses 3: The moderating (i.e., spillover) effect of community education on the association between individual education and contraceptive knowledge, when controlling for other individual and community factors

^e^ Model 5 tests Hypotheses 4a and 4b: The gender difference in the moderating effect of community education on the association between individual education and contraceptive knowledge, when controlling for other individual and community factors

^f^ Variable is mean centered

Model 2 tests of the association between individual education (Hypothesis 1), community education (Hypothesis 2), and contraceptive knowledge—it includes individual education, community education, and gender. Each additional year of education is associated with a 0.130 point increase in contraceptive knowledge, when adjusting for community education and gender. A one-year increase in the average education of the community is associated with a 0.065 point increase in contraceptive knowledge when adjusting for individual education and gender. Finally, men have 0.507 fewer contraception knowledge points than women, when adjusting for individual and community education. Model 3 similarly tests Hypothesis 1 and 2, while adjusting for additional individual- and community-level covariates. In Model 3, the slope for community education changed little while the slope for individual education increased to 0.161 and the slope for gender decreased to -0.406, but there was no change in the direction of association when adjusting for other individual and community factors.

Model 4 further includes an interaction term between individual and community education to test whether community education moderates the association between individual education and contraceptive knowledge (Hypothesis 3). The positive association between individual education and contraceptive knowledge is strongest in communities with lower education, but becomes weaker as community education increases. There is a 0.159 point increase in contraceptive knowledge for each additional year of individual education, at the mean years of community education, and when adjusting for all other variables in the model. However, this association between individual education and contraceptive knowledge becomes 0.006 points less positive for each additional 1-year increase in community education.

**Fig. 3:**
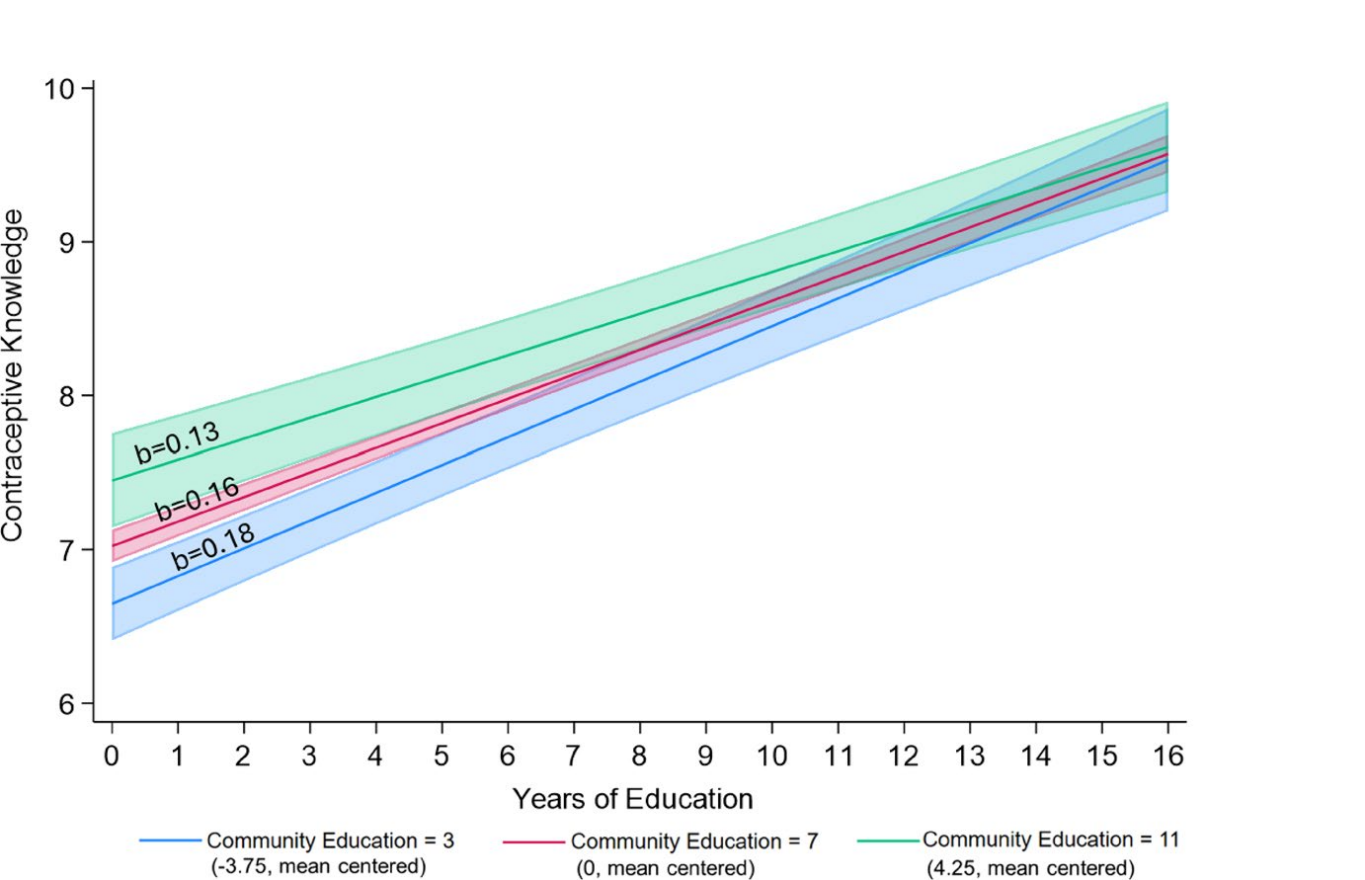
Predicted association between individual education and knowledge of contraceptive methods in communities with 3, 7, and 11 years of education on average^a,b^. *Notes:* ^a^ Predicted margins with 95% confidence intervals are calculated based on results from Table 3, Model 4 with travel and internet use included at the mean of all control variables: gender, age, marital status, household wealth, paid employment, % distance to a health clinic as a barrier to care, % of community with paid employment, mean community age, % of community with below average wealth, and rural household. ^b^ Three years of education represents the end of lower primary schooling and approximately two standard deviations below the mean for community education. Seven years of education represents the end of upper primary schooling and is approximately the mean for community education. Eleven years of education represents the end of secondary schooling and approximately two standard deviations above the mean for community education

Figure 3 plots the association between individual education (horizontal axis) and contraceptive knowledge (vertical axis), when community education takes the values of 3, 7, or 11 years (i.e., − 3.75, 0, and 4.25 when mean centered), net of other covariates from Model 4. We include slopes for the gradient of individual education with contraceptive knowledge at each level of community education, which demonstrates the magnitude of these effects. For each additional year of individual education, contraceptive knowledge increases by 0.18 (= 0.159 − 0.006 × − 3.75) points in communities with an average of 3 years of schooling (i.e., − 3.75 when mean centered), 0.16 (= 0.159 − 0.006 × 0) points in communities with an average of 7 years of schooling (i.e., 0 when mean centered), and 0.13 (= 0.159 − 0.006 × 4.25) in communities with an average of 11 years of schooling (i.e., 4.25 when mean centered). To understand the magnitude of these effects, we compare gaps between those with one year of education versus those who completed secondary schooling (i.e., 11 years of education) in communities with different education levels. Among those living in communities with an average of three years of education, there is a difference of approximately two points in contraceptive knowledge between those with one versus 11 years of education. In contrast, in communities with an average of 11 years of education, contraceptive knowledge is approximately 1.5 points lower among those with one year of education when compared to those with 11 years of education.

Model 5 builds on Model 4 and additionally includes three-way interactions between individual education, community education, and gender, including the two-way interaction terms for gender by individual education and gender by community education. This allows us to test whether the moderating effect of community education on the association between individual education and contraceptive knowledge from Model 4 differs for men and women (Hypotheses 4a and 4b). We find a significant three-way product term at the 95% confidence level (*p* = 0.015), suggesting that there is a difference between men and women in the effect of community education on the relationship between individual education and contraceptive knowledge. For each additional year of individual education there is a 0.142 increase in contraceptive knowledge, for women, at the mean years of community education, and when adjusting for all other variables in the model. The association between individual education and contraceptive knowledge among women becomes 0.00047 points more positive for each additional one-year increase in community education. For men, each additional year of individual education is associated with a 0.177 (= 0.142 + 0.035) increase in contraceptive knowledge, at the mean years of community education, and adjusting for all other variables. Further, among men, the association between individual education and contraceptive knowledge becomes − 0.013 (= 0.00047 − 0.013) points less positive for each additional one-year increase in community education.

**Fig. 4:**
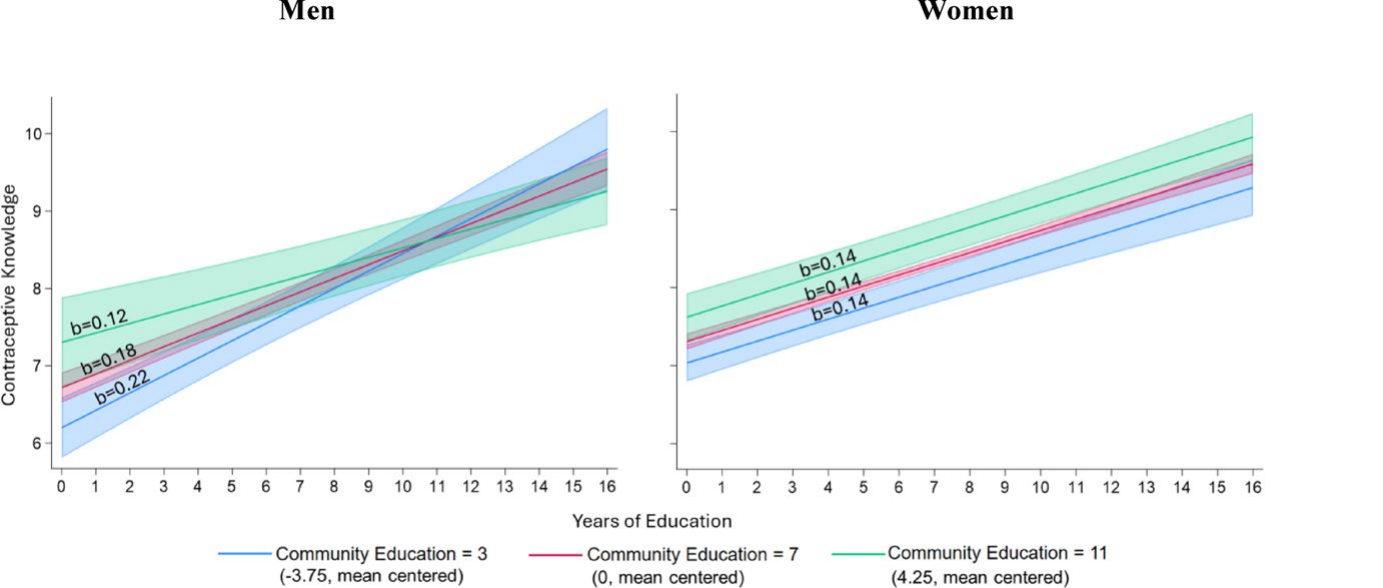
Predicted association between individual education and knowledge of contraceptive methods for men and women in communities with 3, 7, and 11 years of education on average^a,b^. *Notes:* ^a^ Predicted margins with 95% confidence intervals are calculated separately for men and women based on results from Table 3, Model 5 at the mean of all control variables: age, marital status, household wealth, paid employment, % distance to a health clinic as a barrier to care, % of community with paid employment, mean community age, % of community with below average wealth, and rural household. ^b^ Three years of education represents the end of lower primary schooling and approximately two standard deviations below the mean for community education. Seven years of education represents the end of upper primary schooling and is approximately the mean for community education. Eleven years of education represents the end of secondary schooling and approximately two standard deviations above the mean for community education

To facilitate the interpretation of these results, we plot the interaction between individual education and community education in relation to contraceptive knowledge in Fig. 4, with separate panels for men and women. The gradient of each slope is also provided in each panel. For men the association between individual education and contraceptive knowledge is much stronger in communities with lower average education, and much weaker in communities with a higher average education. In contrast, for women there is little difference in the association between individual education and contraceptive knowledge in communities with lower versus higher education levels.

## Discussion and Conclusion

Investments in educational attainment are often hailed as a powerful strategy for improving health outcomes throughout sub-Saharan Africa. Although existing research often theorizes that sexual and reproductive health knowledge explains the association between formal education and sexual and reproductive health outcomes, limited research directly examines the connection between formal educational attainment and sexual and reproductive health knowledge at the population level (Agüero & Bharadwaj, 2014; Basu, 2002; Psaki et al., 2019). Our study offers new insights about the connection between education and sexual and reproductive health knowledge by drawing on human capital and social learning theories to conceptualize and then model how individual and community education levels might jointly shape sexual and reproductive health knowledge. Further, we extend prior research that focuses primarily on women, by theorizing and testing gender differences in the associations between individual and community education and sexual and reproductive health knowledge.

### Individual Education

Our finding that individuals with more education have more accurate sexual and reproductive health knowledge is consistent with hypothesis 1, extant theory, and prior evidence (see Models 2 and 3 in Tables 2 and 3) (Agüero & Bharadwaj, 2014; Blackstone et al., 2017). This finding holds even while adjusting for community education and other factors such as wealth and access to health clinics (see Model 3). In alignment with human capital theories, our results support the premise that education confers Ugandans with internally held skills and resources that they can use to acquire health knowledge and to protect them from inaccurate health information (Mirowsky & Ross, 2005). Our research gives some credence to public health interventions that operate through increasing individual access to formal schooling. One well-known example of such an intervention in east Africa is PEPFAR’s DREAMS project, which funnels HIV funding into programs to improve young women’s access to formal schooling as a means to reduce HIV risk (Birdthistle et al., 2018).

### Community Education

In support of hypothesis 2, we find that average levels of community education are significantly and positively associated with sexual and reproductive health knowledge (see Models 2 and 3 in Tables 2 and 3). Our results are consistent with findings from both high and low resource countries that show that living in more highly educated communities is predictive of a variety of positive health outcomes for the more and less educated alike (Arcaya et al., 2016; Pamuk et al., 2011). One frequently cited mechanism for explaining such associations is that there is greater access to health care resources—often facilitated by the pressures from wealthier neighbors—within more educated neighborhoods, which may be particularly relevant for more distal health outcomes, such as mortality, in contexts in the global north (Boylan & Robert, 2017; Diez Roux & Mair, 2010; Phelan et al., 2010). In contrast, our findings in Uganda show that for an individual’s health *knowledge*, the education of community members has a separate and direct influence even when controlling for other community-level factors such as wealth and distance to the health clinic (see Model 3). Community levels of education in Uganda improve individual's sexual and reproductive health knowledge, even if that sexual and reproductive health knowledge is not gained directly through an individual’s own schooling. In order to target limited health and education resources among those with the greatest need, our findings suggest that policy makers in east Africa should prioritize scaling sexual and reproductive health knowledge interventions in less-educated communities.

### Intersection of Individual and Community Education

In support of hypothesis 3, we find that the association between individual education and sexual and reproductive health knowledge is stronger in less educated communities and weaker in more educated communities. As seen in Figs. 1 and 3, three key findings demonstrate that sexual and reproductive health knowledge is being shared within communities via social learning, and that individuals with more education are less susceptible than their less educated neighbors to the sexual and reproductive health knowledge circulating in their communities—simultaneously demonstrating the importance of human capital. First, in support of human capital theories, we show that those with secondary or higher schooling have similarly high sexual and reproductive health knowledge across all communities. Second, those who did not complete primary school have low levels of sexual and reproductive health knowledge when living in communities with low levels of education. The first and second points, when taken together, indicate that greater individual education simultaneously facilitates the acquisition of more accurate sexual and reproductive health information and shields individuals against the limited or inaccurate sexual and reproductive health information circulating in a community. Third, however, in more educated communities, those who did not complete primary school have higher levels of sexual and reproductive health knowledge—more similar to their more educated neighbors and less similar to those with similarly low education in less educated communities. Taken together, the second and third points demonstrate a spillover effect—those with less education tend to acquire the predominant sexual and reproductive health knowledge that is circulating in their communities.

These findings extend prior literature about the importance of social networks for the diffusion of sexual and reproductive health knowledge in sub-Saharan Africa (Kohler et al., 2000; Rewley et al., 2020), and suggest that education shapes these social learning processes. Human capital and social learning theories work in tandem—formal schooling benefits the health knowledge of the individual and structures the health knowledge circulating in communities. In sensitivity analyses (see online appendix Tables S2 and S3) we re-ran models 3–5 including internet use and travel in the past year—potential confounding factors. The overall patterns we identified were robust to this change, with a slight increase in the observed spillover effect for contraceptive knowledge (see also online appendix Figs. S5 and S6).

Our results suggest the potential for exacerbated education-related health disparities within the lowest educated communities, with attenuation of those disparities in more highly educated communities. In the least educated communities, those with no education would acquire one less piece of accurate information about how to avoid HIV infection, and awareness of two fewer methods to avoid an unwanted pregnancy, compared with those who completed secondary schooling. In less educated communities, these education disparities could have important implications for a less educated individual’s ability to accurately make decisions regarding their HIV risk and/or effectively prevent unintended pregnancies in a manner that best suits their needs. However, in more highly educated communities, the more minimal gaps in sexual and reproductive health knowledge across levels of individual education are likely to have a more minimal effect on individuals’ abilities to engage in HIV and pregnancy prevention. In the most highly educated communities, the difference between those with no versus completed secondary schooling represents less than one piece of HIV prevention information and less than two types of contraception awareness. We note, however, that knowledge alone is insufficient if individuals lack the resources to enact that knowledge, including access to a full range or contraceptive methods and HIV prevention tools such as condoms.

Our support for social learning processes that pattern educational associations with sexual and reproductive health knowledge in Uganda have two key policy implications. First, efforts in the region to increase education access may have the greatest returns to sexual and reproductive health knowledge—and possibly health more broadly—in the communities with lowest education, rather than among the least educated individuals in more highly educated communities. In sub-Saharan Africa, the near universal expansion of free primary education has led to recent calls for reaching those who have not yet entered the educational system—the hardest to reach families and individuals within a community (Grant, 2015; United Nations, 2023). While improving educational access for everyone would likely provide many benefits beyond sexual and reproductive health knowledge, our findings suggest that the least educated individuals in more highly educated communities may still benefit from the education and sexual and reproductive health knowledge in the broader community. Thus, in settings like Uganda with limited education and health resources, efforts to increase education access specifically among the least educated individuals living in communities with low average levels of education are likely to have the largest population health impact in the long run.

Second, our findings suggest the value of both prioritizing sexual and reproductive health interventions in lower educated communities, and doing so in a manner that specifically addresses education disparities in sexual and reproductive health knowledge within those specific communities. Interventions that use the principles of community capacity development—building on existing community resources—can intentionally capitalize on the social learning processes we observed (Rewley et al., 2020). Examples of such interventions in southern and eastern Africa include community health worker programs and community-based reading clubs wherein community members interact and support each other with their knowledge and skills (Mwai et al., 2013; Smith et al., 2006). We note that this recommendation is not simply for a replication of the widespread ‘community health promotion’ interventions that already exist in Uganda. While those interventions hold value, our findings—alongside previous research on social learning—suggest that interventions should directly address the knowledge disseminated through more organic social interactions. By applying those principles, public health practitioners in eastern Africa can influence the conditions that support the diffusion of accurate and complete sexual and reproductive health knowledge throughout the population. In the future, more qualitative work to inform intervention design is needed to understand how individuals from different educational strata are interacting and sharing sexual and reproductive health knowledge, including understanding how less accurate information is sourced and circulated.

### Gender Differences: Intersection of Individual and Community Education

Our findings did not support our hypotheses 4a and 4b. For men, hypothesis 4a predicted strong positive associations between individual education and sexual and reproductive health knowledge across all communities in Uganda. However, we observed a different pattern: there was a strong positive association between individual education and sexual and reproductive health knowledge in lower education communities, but that association became weaker as community education increased. These results suggest that we under-estimated the proportion of Ugandan men’s sexual and reproductive health knowledge that is gained through social learning processes in their home communities. These findings are consistent with other evidence from the region that demonstrates that men’s contraceptive choices and HIV decision making are strongly influenced by social interactions (Behrman et al., 2002; Coursey et al., 2023).

Perplexingly, our results for men reflect the pattern that we had expected to see for women in our hypothesis 4b. The findings for women were inconsistent across our outcome measures. Hypothesis 4b predicted that the association between women’s individual education and sexual and reproductive health knowledge would be stronger in lower educated communities and weaker as community education increased. Our results for HIV knowledge support hypothesis 4b, which suggests that women, similar to men, accrue HIV knowledge through social learning. In contrast, for women’s contraceptive knowledge, individual education was a strong, positive predictor in both lower and higher educated communities—a pattern we originally expected to see only for men. Our findings indicate that, across Ugandan communities, women receive a smaller proportion of their contraceptive knowledge through social learning processes and rely more on their own education-related skills and resources to obtain information about contraceptive options.

The divergent patterns for women’s contraceptive knowledge and HIV knowledge may be explained by Ugandan women’s health service utilization and the nature of the survey questions we used. For contraception, we assessed the number of specific modern family planning options that respondents knew about, which is information they are likely to receive during individual health care services. In contrast, our measure of HIV knowledge is more connected to ideas that can easily circulate in the community, and which are less strongly tied to information and technology gained through clinical services. Because women with more education are more likely to use reproductive health services, this may explain why women’s individual education is also strongly predictive of their knowledge of specific contraceptive methods across communities (Chama-Chiliba & Koch, 2013; Larsson & Stanfors, 2014; Okedo-Alex et al., 2019).

In the context of Uganda, different approaches may be warranted for addressing men’s and women’s contraceptive knowledge. Efforts to increase men’s contraceptive knowledge may yield the greatest benefits in lower educated communities. Such interventions could complement on-going efforts to engage more men in family planning services (Beia et al., 2021). In support of policy goals to reduce unmet needs for family planning options in sub-Saharan Africa (Langer et al., 2015), interventions might target educational disparities in contraceptive knowledge for women across all communities. Our findings go beyond a story about limited contraceptive availability in lower-resource Ugandan communities leading to limited contraceptive knowledge among women in those same communities. Even in areas where there is more education and likely a greater range of contraceptive options available, less educated Ugandan women are less aware of those options than their more educated neighbors. Education disparities in awareness of *available* options may constrain less educated women’s autonomy in choosing the method that is best for them. Interventions outside of the health clinic are needed to ensure that less educated women are fully aware of their family planning options.

### Strengths and Limitations

Our study has several key strengths. First, we extend prior research by focusing on sexual and reproductive health knowledge—a mechanism that is theorized to explain the relationship between education and sexual and reproductive health outcomes, but that is seldom modeled directly. Our findings inform how education and public health policies can efficiently and strategically target sexual and reproductive health knowledge, by jointly considering which communities and individuals have the greatest need for education access and sexual and reproductive health promotion interventions. Second, we use comprehensive measures of contraceptive and HIV knowledge, in contrast to studies that rely on basic, binary measures of knowing versus not knowing about family planning or HIV. In a context where basic knowledge is widespread, gradients in comprehensive sexual and reproductive health knowledge may be more predictive of individual’s abilities to prevent unwanted sexual and reproductive health outcomes, making our measures particularly relevant to future research and theory development. Third, unlike much existing research in sub-Saharan Africa, we include men in our individual and community measures, allowing us to see the influence of education for sexual and reproductive health knowledge among all community members, and not just women. Our findings for contraceptive knowledge show divergent patterns for men and women, and reinforce the need for research that specifically examines gender disparities in acquiring and utilizing sexual and reproductive health knowledge.

Finally, we looked at two forms of sexual and reproductive health knowledge which allowed us make a key theoretical insight: the diffusion of sexual and reproductive health information will likely operate differently for different types of knowledge. In situations where there are strong individual educational disparities in accessing formal healthcare and where sexual and reproductive health information is tightly connected to those clinical settings, it is likely that a greater portion of individual’s sexual and reproductive health knowledge will be determined by their individual education, with less of an effect from social learning processes.

Despite these strengths our study has some key limitations. First, our data are cross-sectional, limiting our ability to assess the temporal effect of educational attainment on the acquisition of sexual and reproductive health knowledge. Nevertheless, we are still able to demonstrate key differences in existing sexual and reproductive health knowledge among people and communities with different levels of education. Second, while our models adjust for key individual and community factors that are most likely to influence the education-sexual and reproductive health knowledge relationship, some potential confounders are unavailable in our data. Key among these are the other resources in a given community, such as civil society organizations, micro-finance institutions, and less tangible community social support systems, which could support the dissemination of more accurate sexual and reproductive health information. As these community resources tend to coincide with greater educational opportunities in communities, it is possible that the associations we observe between community education and sexual and reproductive health knowledge, are less a reflection of the influence of community members’ education and more of a reflection of the influence of living in better resourced areas. That said, we believe a large portion of this variance in community resources is captured in our models by controlling for distance to the health clinic and rurality, allowing us to more precisely ascertain the influence of community education alone.

Third, our measures of sexual and reproductive health knowledge, while useful for the purposes of this analysis, do not fully represent all domains of sexual and reproductive health knowledge. For example, other dimensions of HIV knowledge could include the importance of early and consistent HIV treatment; or additional knowledge about family planning may include an understanding of the reproductive cycle or how to effectively use condoms. It is unclear if the patterns we observed for knowledge about HIV transmission and contraceptive options would persist for other forms of sexual and reproductive health knowledge or for other types of health knowledge more broadly. Finally, we recognize that our use of the enumeration area “clusters” to define community may not be the most accurate reflection of the social circles that are most relevant in Ugandan’s lives.

## Conclusion

Our study provides new insight into the positive role of formal education in shaping how people obtain and exchange sexual and reproductive health knowledge. Our findings demonstrate that living in more educated communities in Uganda reduces educational disparities in sexual and reproductive health knowledge, but living in less educated communities magnifies those disparities. In the context of limited resources for education and public health, policy makers could focus efforts to increase sexual and reproductive health knowledge among the less educated living in lower education communities. Even as the Sustainable Development Goal #4 emphasizes quality education *for all*, our findings suggest that not everyone needs to achieve the same level of education for the broader community to benefit. In short, our findings offer optimism that even when not everyone is educated to the same extent, increasing education and accurate knowledge in the least educated communities could have long-term population health benefits.

## Supplementary Information

Below is the link to the electronic supplementary material.

Supplementary file 1 (DOCX 1387 kb)

## Data Availability

All data for this research are intended to be publicly available via the Demographic Health Surveys website: https://dhsprogram.com/methodology/survey/survey-display-504.cfm, or can be reviewed by contacting the corresponding author. Additionally, because we conducted a community-level weighting process (described in the Methods section), we drew on household and community estimates from the 2014 Uganda Census, which is also publicly available: https://www.ubos.org/wp-content/uploads/publications/03_20182014_National_Census_Main_Report.pdf. Statistical code for analyses is available from the corresponding author upon request.

## Footnotes

1 This question is not asked of men in the DHS.

2 All households within a cluster/community received the same designation of rural or urban.
