# Supplementary material for: Community and Individual Education Influences on Sexual and Reproductive Health Knowledge in Uganda: A Human Capital and Social Learning Perspective
Source: Popul Res Policy Rev. 2025 Jun 17;44(4):38. doi: 10.1007/s11113-025-09958-y (PMC12174206; doi:10.1007/s11113-025-09958-y)
Supplement: Supplementary file 1 — (DOCX 1387 kb) [file 11113_2025_9958_MOESM1_ESM.docx]

**Online Appendix**

**
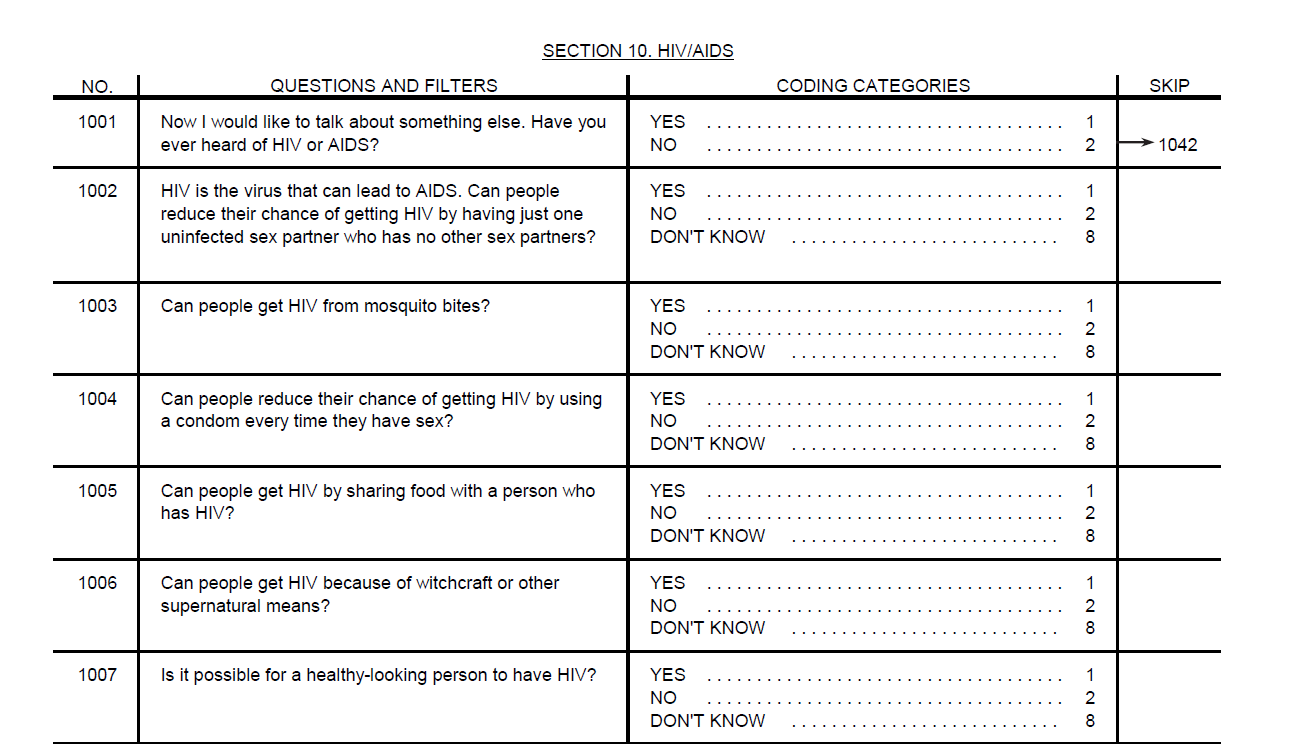
**

**Figure S1.**

*Demographic Health Survey Items Used for Key Outcome Measures: HIV Knowledge*

| *Source.* Uganda Bureau of Statistics (UBOS) and ICF. 2018. *Uganda Demographic and Health Survey 2016,* p. 538, Appendix E. Kampala, Uganda and Rockville, Maryland, USA: UBOS and ICF. |
| --- |


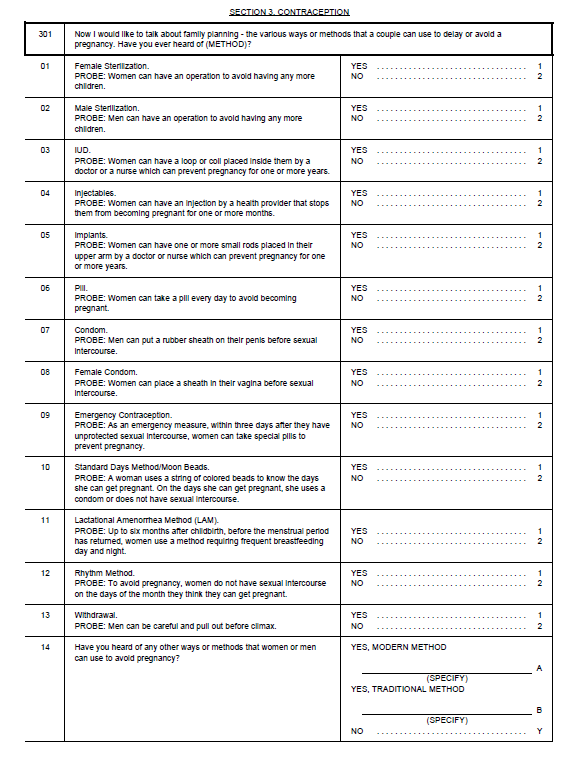


**Figure S2.**

*Demographic Health Survey Items Used for Key Outcome Measures: Contraceptive Knowledge*

| *Source.* Uganda Bureau of Statistics (UBOS) and ICF. 2018. *Uganda Demographic and Health Survey 2016,* p. 492, Appendix E. Kampala, Uganda and Rockville, Maryland, USA: UBOS and ICF. |
| --- |

**Table S1.**

*Percentage Distribution of DHS Uganda 2016 Additional Sample Characteristics for Travel and Internet Use, Weighted, by Gender*

|  | **Total** | **Women** | **Men** | **p-value^a^** |
| --- | --- | --- | --- | --- |
| Travel: Any nights away from home in past year (%) | 51.8 | 52.9 | 50.7 | 0.0473 |
| Internet Exposure: At least weekly internet use (%) | 11.9 | 6.9 | 16.8 | 0.000 |

*Note*. ^a^ Differences between men and women were tested using Pearson’s chi-square.

**Table S2.**

*Multilevel Linear Regression Models of Knowledge of HIV Prevention and Transmission Including Travel and Internet Use, Fixed and Random Effects^a,b^*

|  | ***Model 3^c^*** | ***Model 4^d^*** | ***Model 5^e^*** |
| --- | --- | --- | --- |
| **Fixed Effects** |  |  |  |
|  |  |  |  |
| **Key Predictor Variables** |  |  |  |
| Individual education | 0.062^***^  (0.003) | 0.060^***^  (0.003) | 0.058^***^  (0.003) |
| Community education^f^ | 0.028^**^  (0.010) | 0.080^***^  (0.013) | 0.069^***^  (0.014) |
| Gender (Male=1) | -0.090^***^  (0.024) | -0.092^***^  (0.024) | -0.093^*^  (0.047) |
|  |  |  |  |
| **Interaction terms** |  |  |  |
| Individual education × Community education^f^ | — | -0.008^***^  (0.001) | -0.007^***^  (0.001) |
| Individual education × Gender | — | — | 0.004  (0.005) |
| Community education^f^ × Gender | — | — | 0.053^*^  (0.026) |
| Individual education × Community education^f^ × Gender | — | — | -0.003  (0.002) |
|  |  |  |  |
| **Individual-level control variables** |  |  |  |
| Age^f^ | 0.006^***^  (0.001) | 0.006^***^  (0.001) | 0.006^***^  (0.001) |
| Marital status (Ever married=1) | 0.173^***^  (0.026) | 0.172^***^  (0.026) | 0.172^***^  (0.026) |
| Wealth (quintile) |  |  |  |
| *Poorest (ref)* | — | — | — |
| *Poor* | 0.076^*^  (0.034) | 0.074^*^  (0.034) | 0.073^*^  (0.034) |
| *Average* | 0.124^**^  (0.041) | 0.122^**^  (0.041) | 0.122^**^  (0.041) |
| *Rich* | 0.097^*^  (0.045) | 0.095^*^  (0.045) | 0.095^*^  (0.045) |
| *Richest* | 0.171^**^  (0.052) | 0.170^**^  (0.052) | 0.171^**^  (0.052) |
| Employment (Paid employment in past year=1) | 0.010  (0.022) | 0.011  (0.022) | 0.010  (0.022) |
| Travel (Any nights away from home in past year=1) | 0.057^**^  (0.021) | 0.057^**^  (0.021) | 0.056^**^  (0.021) |
| Internet exposure (At least weekly internet use=1) | 0.200^***^  (0.031) | 0.205^***^  (0.031) | 0.201^***^  (0.032) |
|  |  |  |  |
| **Community-level control variables** |  |  |  |
| % Distance to clinic as a barrier in community | 0.160^**^  (0.054) | 0.155^**^  (0.054) | 0.155^**^  (0.054) |
| % Employed in community | 0.082  (0.089) | 0.087  (0.089) | 0.087  (0.089) |
| Mean Age in community | < -0.001  (0.007) | -0.001  (0.007) | < -0.001  (0.007) |
| % Households with less than average wealth in community | -0.051  (0.069) | -0.047  (0.069) | -0.053  (0.069) |
| Rural household (Rural=1) | 0.013  (0.037) | 0.007  (0.037) | 0.009  (0.037) |
| **Intercept** | 5.356^***^  (0.199) | 5.392^***^  (0.198) | 5.396^***^  (0.198) |
|  |  |  |  |
| **Random effects** |  |  |  |
| Random intercept | 0.249^***^  (0.016) | 0.239^***^  (0.015) | 0.240^***^  (0.015) |
| Random slope for individual education | 0.004^***^  (< 0.001) | 0.004^***^  (< 0.001) | 0.003^***^  (< 0.001) |
| Covariance between the random slope for education and the random intercept | -0.024^***^  (0.002) | -0.022^***^  (0.002) | -0.023^***^  (0.002) |
| Random slope for gender | 0.391^***^  (0.032) | 0.391^***^  (0.032) | 0.381^***^  (0.030) |
| Covariance between the random slope for gender and the random intercept | -0.037^*^  (0.016) | -0.045^**^  (0.016) | -0.042^**^  (0.015) |
| Covariance between the random slope for education and the random slope for gender | -0.009^***^  (0.002) | -0.008^***^  (0.002) | -0.008^***^  (0.002) |
| Residual Variance | 0.908^***^  (0.024) | 0.908^***^  (0.024) | 0.908^***^  (0.024) |

*Notes*. ^†^ *p* < 0.10, ^*^ *p* < 0.05, ^**^ *p* < 0.01, ^***^ *p* < 0.001

^a^ Coefficients with standard errors in parentheses

^b^ See Table 2 in the main text for Models 1 and 2.

^c^ Model 3 tests Hypotheses 1 and 2: The associations between HIV knowledge and education at the individual and community levels, when controlling for other individual and community factors.

^d^ Model 4 tests Hypotheses 3: The moderating (i.e., spillover) effect of community education on the association between individual education and HIV knowledge, when controlling for other individual and community factors.

^e^ Model 5 tests Hypotheses 4a and 4b: The gender difference in the moderating effect of community education on the association between individual education and HIV knowledge, when controlling for other individual and community factors.

^f^ Variable is mean centered.

**
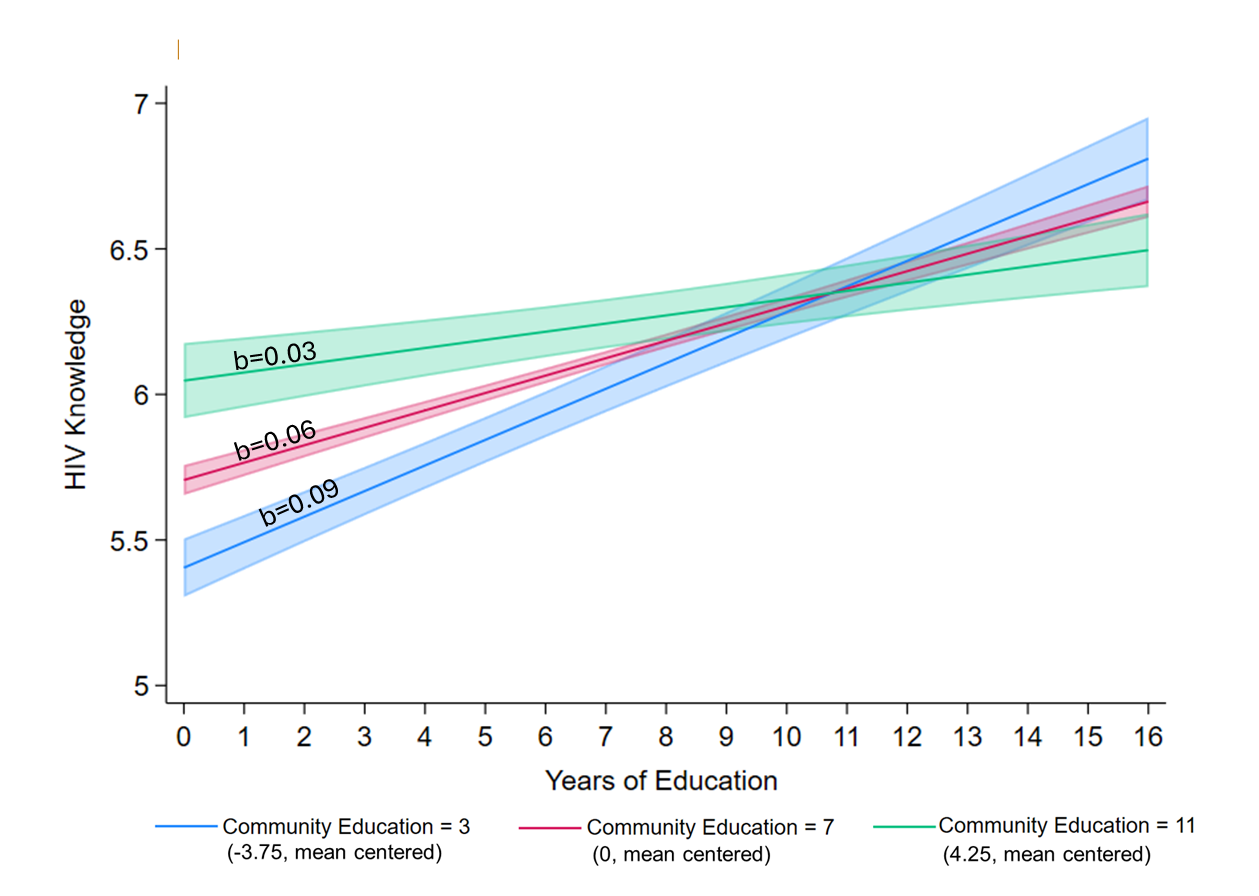
**

**Figure S3.**

*Predicted Association between Individual Education and Knowledge of HIV Prevention and Transmission for Communities with 3, 7, and 11 Years of Education on Average.^a,b^*

| *Notes.*  ^a^ Predicted margins with 95% confidence intervals are calculated based on results from Table S2, Model 4 with travel and internet use included at the mean of all control variables: gender, age, marital status, household wealth, paid employment, % distance to a health clinic as a barrier to care, % of community with paid employment, mean community age, % of community with below average wealth, and rural household.  ^b^ Three years of education represents the end of lower primary schooling and approximately two standard deviations below the mean for community education. Seven years of education represents the end of upper primary schooling and is approximately the mean for community education. Eleven years of education represents the end of secondary schooling and approximately two standard deviations above the mean for community education. |
| --- |

| **Men** | **Women** |
| --- | --- |


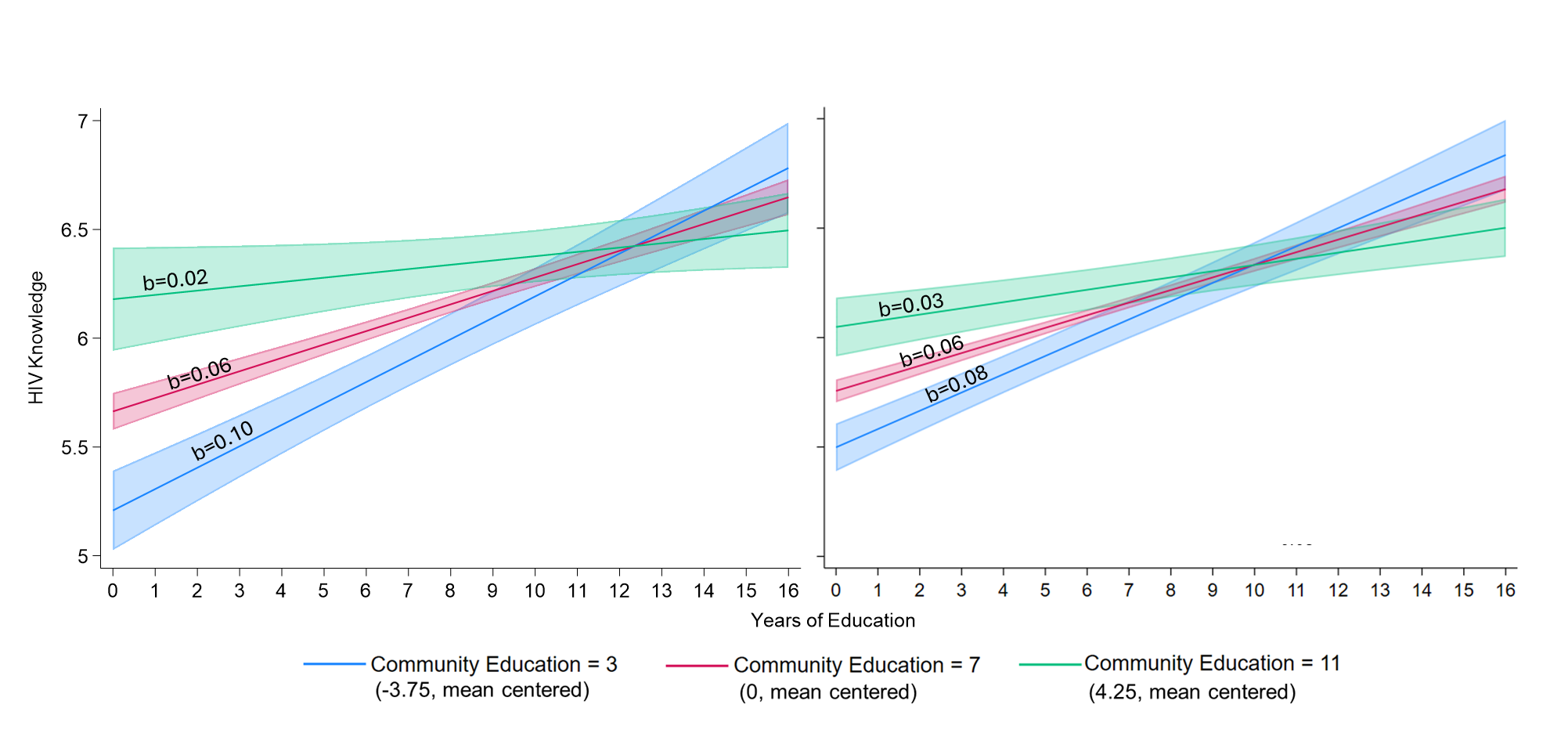


**Figure S4.**

*Predicted Association between Individual Education and Knowledge of HIV Prevention and Transmission for Men and Women in Communities with 3, 7, and 11 Years of Education on Average.^a, b^*

| *Notes.*  ^a^ Predicted margins with 95% confidence intervals are calculated separately for men and women based on results from Table S2, Model 5 at the mean of all control variables: age, marital status, household wealth, paid employment, % distance to a health clinic as a barrier to care, % of community with paid employment, mean community age, % of community with below average wealth, and rural household.  ^b^ Three years of education represents the end of lower primary schooling and approximately two standard deviations below the mean for community education. Seven years of education represents the end of upper primary schooling and is approximately the mean for community education. Eleven years of education represents the end of secondary schooling and approximately two standard deviations above the mean for community education. |
| --- |

**Table S3.**

*Multilevel Linear Regression Models of Knowledge of Contraceptive Methods Including Travel and Internet, Fixed and Random Effects^a,b^*

|  | **Model 3^c^** | **Model 4^d^** | **Model 5^e^** |
| --- | --- | --- | --- |
| **Fixed Effects** |  |  |  |
|  |  |  |  |
| **Key Predictor Variables** |  |  |  |
| Individual education | 0.142^***^  (0.006) | 0.138^***^  (0.006) | 0.129^***^  (0.007) |
| Community education^f^ | 0.066^*^  (0.027) | 0.144^***^  (0.032) | 0.114^***^  (0.032) |
| Gender (Male=1) | -0.454^***^  (0.055) | -0.456^***^  (0.055) | -0.540^***^  (0.106) |
|  |  |  |  |
| **Interaction terms** |  |  |  |
| Individual education × Community education^f^ | — | -0.012^***^  (0.003) | -0.006 ^*^  (0.003) |
| Individual education × Gender | — | — | 0.019  (0.013) |
| Community education^f^ × Gender | — | — | 0.056  (0.053) |
| Individual education^f^ × Community education × Gender | — | — | -0.012^*^  (0.005) |
|  |  |  |  |
| **Individual-level control variables** |  |  |  |
| Age^f^ | 0.037^***^  (0.002) | 0.037^***^  (0.002) | 0.037^***^  (0.002) |
| Marital status (Ever married=1) | 1.535^***^  (0.065) | 1.534^***^  (0.065) | 1.533^***^  (0.065) |
| Wealth (quintile) |  |  |  |
| *Poorest (ref)* | — | — | — |
| *Poor* | 0.110  (0.069) | 0.108  (0.069) | 0.106  (0.069) |
| *Average* | 0.142^*^  (0.074) | 0.139^†^  (0.074) | 0.138^†^  (0.074) |
| *Rich* | 0.222^*^  (0.087) | 0.219^*^  (0.088) | 0.219^*^  (0.088) |
| *Richest* | 0.223^*^  (0.113) | 0.222^*^  (0.113) | 0.223^*^  (0.113) |
| Employment (Paid employment in past year=1) | 0.386^***^  (0.044) | 0.386^***^  (0.044) | 0.386^***^  (0.044) |
| Travel (Any nights away from home in past year=1) | 0.326^***^  (0.044) | 0.326^***^  (0.044) | 0.323^***^  (0.044) |
| Internet exposure (At least weekly internet use=1) | 0.719^**^  (0.081) | 0.726^***^  (0.081) | 0.712^***^  (0.082) |
|  |  |  |  |
| **Community-level control variables** |  |  |  |
| % Distance to clinic as a barrier in community | 0.545^***^  (0.147) | 0.533^***^  (0.146) | 0.533^***^  (0.146) |
| % Employed in community | -0.352  (0.236) | -0.339  (0.236) | -0.363  (0.236) |
| Mean Age in community | -0.001  (0.018) | -0.002  (0.018) | -0.002  (0.018) |
| % Households with less than average wealth in community | -0.313  (0.194) | -0.305  (0.194) | -0.347^†^  (0.193) |
| Rural household (Rural=1) | 0.151^†^  (0.090) | 0.141  (0.090) | 0.179^*^  (0.091) |
| **Intercept** | 5.768^***^  (0.549) | 5.826^***^  (0.546) | 5.876^***^  (0.545) |
|  |  |  |  |
| **Random Effects** |  |  |  |
| Random intercept | 1.192^***^  (0.085) | 1.166^***^  (0.083) | 1.185^***^  (0.084) |
| Random slope for individual education | 0.016^***^  (0.001) | 0.015^***^  (0.001) | 0.015^***^  (0.001) |
| Covariance between the random slope for education and the random intercept | -0.107^***^  (0.009) | -0.104^***^  (0.009) | -0.105^***^  (0.008) |
| Random slope for gender | 2.260^***^  (0.175) | 2.260^***^  (0.175) | 2.199^***^  (0.171) |
| Covariance between the random slope for gender and the random intercept | 0.113  (0.074) | 0.121  (0.075) | 0.114  (0.075) |
| Covariance between the random slope for education and the random slope for gender | -0.037^***^  (0.010) | -0.039^***^  (0.010) | -0.037^***^  (0.010) |
| Residual Variance | 3.604^***^  (0.070) | 3.604^***^  (0.070) | 3.602^***^  (0.070) |

*Notes*. ^†^ *p* < 0.10, ^*^ *p* < 0.05, ^**^ *p* < 0.01, ^***^ *p* < 0.001

^a^ Coefficients with standard errors in parentheses

^b^ See Table 3 in the main text for Models 1 and 2.

^c^ Model 3 tests Hypotheses 1 and 2: The associations between contraceptive knowledge and education at the individual and community levels, when controlling for other individual and community factors.

^d^ Model 4 tests Hypotheses 3: The moderating (i.e., spillover) effect of community education on the association between individual education and contraceptive knowledge, when controlling for other individual and community factors.

^e^ Model 5 tests Hypotheses 4a and 4b: The gender difference in the moderating effect of community education on the association between individual education and contraceptive knowledge, when controlling for other individual and community factors.

^f^ Variable is mean centered.

**
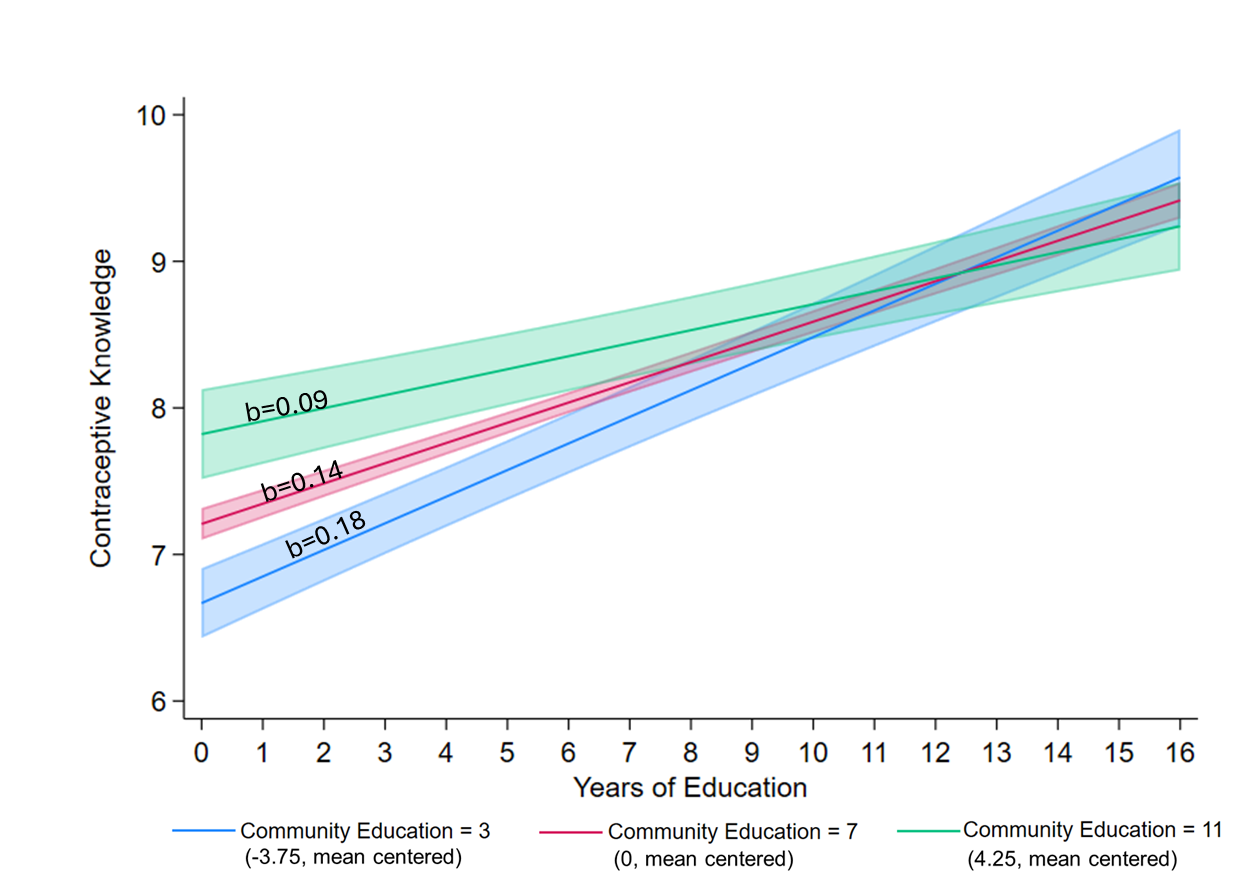
**

**Figure S5.**

*Predicted Association between Individual Education and Knowledge of Contraceptive Methods in Communities with 3, 7, and 11 Years of Education on Average.^a, b^*

| *Notes.*  ^a^ Predicted margins with 95% confidence intervals are calculated based on results from Table S3, Model 4 with travel and internet use included at the mean of all control variables: gender, age, marital status, household wealth, paid employment, % distance to a health clinic as a barrier to care, % of community with paid employment, mean community age, % of community with below average wealth, and rural household.  ^b^ Three years of education represents the end of lower primary schooling and approximately two standard deviations below the mean for community education. Seven years of education represents the end of upper primary schooling and is approximately the mean for community education. Eleven years of education represents the end of secondary schooling and approximately two standard deviations above the mean for community education. |
| --- |

| **Men** | **Women** |
| --- | --- |

**
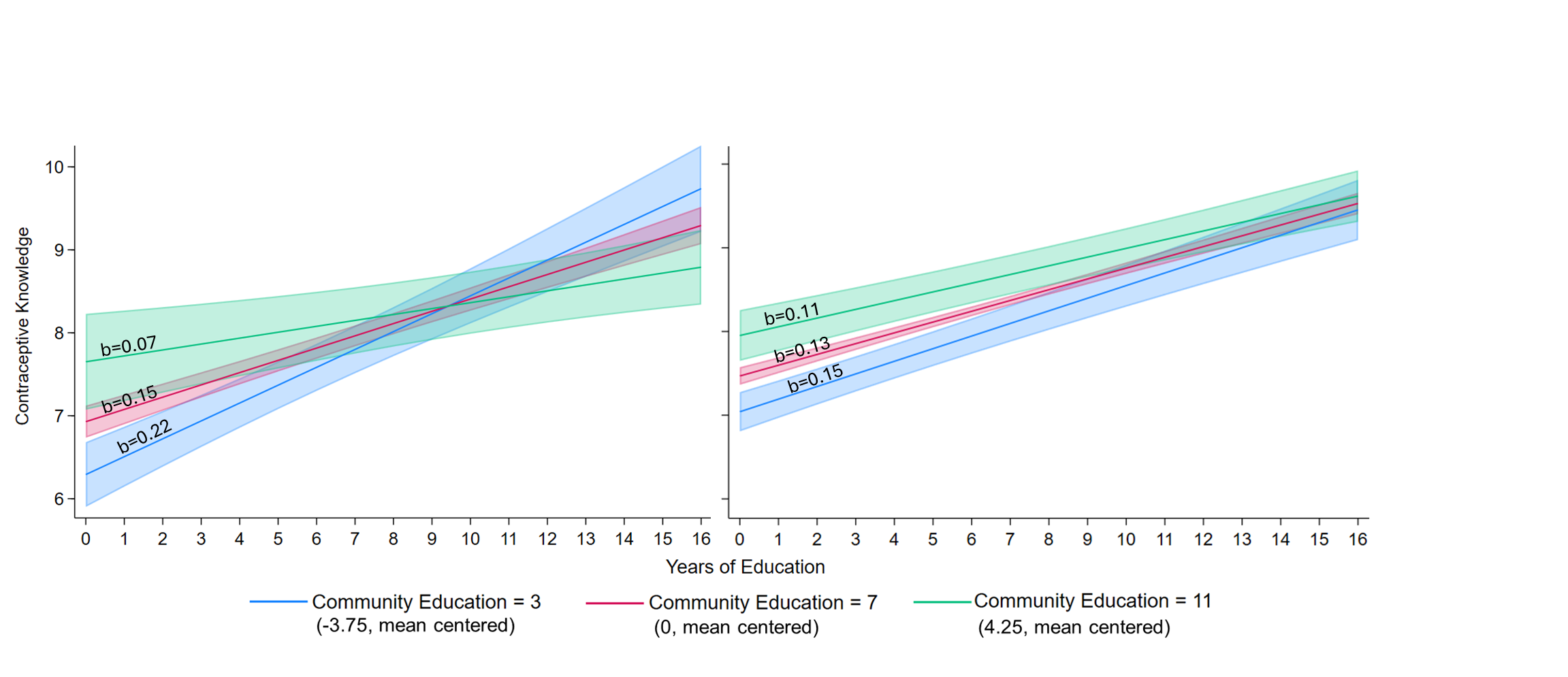
**

**Figure S6.**

*Predicted Association between Individual Education and Knowledge of Contraceptive Methods for Men and Women in Communities with 3, 7, and 11 Years of Education on Average.^a, b^*

| *Notes.*  ^a^ Predicted margins with 95% confidence intervals are calculated separately for men and women based on results from Table S3, Model 5 at the mean of all control variables: age, marital status, household wealth, paid employment, % distance to a health clinic as a barrier to care, % of community with paid employment, mean community age, % of community with below average wealth, and rural household.  ^b^ Three years of education represents the end of lower primary schooling and approximately two standard deviations below the mean for community education. Seven years of education represents the end of upper primary schooling and is approximately the mean for community education. Eleven years of education represents the end of secondary schooling and approximately two standard deviations above the mean for community education. |
| --- |
